# Methods of Model Reduction for Large-Scale Biological Systems: A Survey of Current Methods and Trends

**DOI:** 10.1007/s11538-017-0277-2

**Published:** 2017-06-27

**Authors:** Thomas J. Snowden, Piet H. van der Graaf, Marcus J. Tindall

**Affiliations:** 1 Department of Mathematics and Statistics, University of Reading, Reading, RG6 6AX UK; 2 Certara QSP, University of Kent Innovation Centre, Canterbury, CT2 7FG UK; 3 Leiden Academic Centre for Drug Research, Universiteit Leiden, Leiden, 2333 CC Netherlands; 4 The Institute for Cardiovascular and Metabolic Research (ICMR), University of Reading, Reading, RG6 6AX UK

**Keywords:** Model reduction, Complexity, Systems biology, Mathematical modelling, 34A34, 37N25, 65Y20, 92-08

## Abstract

Complex models of biochemical reaction systems have become increasingly common in the systems biology literature. The complexity of such models can present a number of obstacles for their practical use, often making problems difficult to intuit or computationally intractable. Methods of model reduction can be employed to alleviate the issue of complexity by seeking to eliminate those portions of a reaction network that have little or no effect upon the outcomes of interest, hence yielding simplified systems that retain an accurate predictive capacity. This review paper seeks to provide a brief overview of a range of such methods and their application in the context of biochemical reaction network models. To achieve this, we provide a brief mathematical account of the main methods including timescale exploitation approaches, reduction via sensitivity analysis, optimisation methods, lumping, and singular value decomposition-based approaches. Methods are reviewed in the context of large-scale systems biology type models, and future areas of research are briefly discussed.

**Electronic supplementary material:**

The online version of this article (doi:10.1007/s11538-017-0277-2) contains supplementary material, which is available to authorized users.

## Introduction

Model complexity can be used to refer to a number of specific properties of mathematical models occurring in a range of scientific contexts. It can, for example, be used to refer to models that are overparameterised relative to the volume of collectable data, models that are unintuitable due to their scale, or models that are computationally intractable in magnitude. In each case, complexity presents a barrier to standard tools of model analysis. Methods of model reduction offer one possible approach for dealing with the perennial issue of model complexity by seeking to approximate the behaviour of a model by constructing a simplified dynamical system that retains some degree of the predictive power of the original.

Model reduction has a long history in the mathematical modelling of biological systems; perhaps the most famous example is Briggs and Haldane’s application of the quasi-steady-state approximation (QSSA) for the simplification of a model of the enzyme–substrate reaction (Briggs and Haldane 1925). They demonstrated that a simplifying assumption could take the unsolvable, nonlinear, four-dimensional system of coupled ordinary differential equations (ODEs) that constituted the model, to a single ODE whilst still providing an accurate description of the dynamics for a wide range of possible parameterisations.

The mathematical modelling of biological processes often leads to highly complex systems involving many state-variables and reactions. The relatively recent advent of systems biology, which seeks to model such systems in detail and hence yield a high degree of mechanistic exploratory power, has greatly increased this complexity such that it is now common to encounter models containing hundreds or even thousands of variables (Li et al. 2010).

Even given this rapid increase in complexity, however, concurrent advances in computing power and simulation algorithms may appear to make model reduction a less essential process than it was in the past—it is now possible to accurately and efficiently compute numerical simulations of even highly complex systems where previously some degree of reduction was necessary to understand even the basic dynamical behaviour of many models. Ease of simulation, however, does not necessarily lead to depth of understanding; for a wide range of analyses model complexity can present an insurmountable barrier. Methods of model reduction therefore remain a vital topic and a widely applicable tool in the analysis and modelling of biochemical systems. The methods that will be discussed throughout this paper have been employed for a wide range of purposes in the literature, including to obtain more intuitively understood models, to reduce the number of parameters so as to obtain an identifiable model, to lessen the computational burden of parameter fitting, and to enable the embedding of such systems within agent-based modelling approaches. Here, for example, a researcher may be interested in concurrently modelling a large number of cells comprising a tissue—by employing a reduced description of the individual cells, such a problem may be made more computationally feasible.

Despite the utility of model reduction methods, familiarity is often limited to a small range of methods that can be found in the literature. This review therefore seeks to give an overview of the use and application of model reduction methods in this context.

Such methods are commonly applied within the fields of engineering and control theory, and a number of reviews of methods within these contexts exist (Okino and Mavrovouniotis 1998; Antoulas 2005). Additionally, Radulescu et al. (2012) have reviewed timescale exploitation methods for the reduction of computational biology models, but their work mostly focuses on the fundamental basis of such methods and the potential applicability of model tropicalisation in this context. The aim of this review is therefore to provide a more contextualised and up-to-date overview of such methods, as well as a survey of the current state of the literature, so as to better assess the possible utility of particular model reduction methodologies for application in the field of systems biology.

The broader topic of general model reduction methods is an extensive area of study. To review the entire field would be a challenging undertaking and beyond the scope of this paper. As a result, this review limits itself in the following respects; firstly, the survey of literature is limited only to those methods that have been developed, adapted or applied in the context of biochemical reaction network models. Secondly, it is limited to methods addressing models that are comprised of systems of ODEs. Thirdly, it focuses particularly on those methods that have seen published application within the previous 15 years. Ideally, such methods will be algorithmic, automatable and produce highly accurate, significantly reduced approximations.

By reviewing such a range of literature we are able to separate methods into categories and provide insight into their suitability in addressing certain classes of problems. In the discussion section we provide an overview of methods and their general applicability, collating this information in Table 1 to summarise the suitability of the different methods in the context of particular model properties. It is hoped this can therefore provide guidance to the most appropriate methods currently available for reducing models.

### Problem Outline

Mathematically, this review seeks to address the reduction of large-scale models of biochemical reaction networks represented by high-dimensional systems of (typically nonlinear) ODEs. These are usually informed by sets of interacting chemical equations that can be expressed in the form of ODEs via application of the Law of Mass Action. Such a system of chemical equations is comprised of an *n*-dimensional set of individual species $s_i\in \mathcal {S}$ (the chemical reactants), an *m*-dimensional set of reactions $\mathcal {R}$ describing the interaction and transition of these species, and an associated *m*-dimensional vector of time-invariant kinetic parameters $\varvec{p}\in \mathbb {R}^m$ describing the frequency with which each of the reactions occurs under the assumption that the reactants are well stirred.

The Law of Mass Action then allows the description of the dynamics of these reactants *en masse* such that the model describes the overall change in the molecular concentration of the reactants. To achieve this, the variables $x_i(t) \in \varvec{x}(t)$ are defined to represent the instantaneous concentrations associated with each of the species $s_i \in \mathcal {S}$, such that $\varvec{x}(t): \mathcal {S} \rightarrow \mathbb {R}^n_{\ge 0}$, with *t* an independent variable representing time. It is common notation to use square brackets to represent the instantaneous molecular concentration of a species, hence in this form $x_i(t) = \left[ s_i\right]$. The Law of Mass Action then states that the rate of concentration change due to a given reaction is proportional to the product of the active masses of the reactants each raised to a power equal to their reactant stoichiometric coefficient. Additionally, the coefficient of proportionality is equal to the corresponding kinetic parameter $p_i\in \varvec{p}$. Given this, it is possible to define a vector of reaction rates $\varvec{v}\left( \varvec{x}(t),\varvec{p}\right)$ explicitly describing the rate of molecular concentration change due to each reaction.

To understand how these reaction rates influence the overall dynamics of the system, it is further necessary to account for the overall network structure and to describe how each of the species is involved in each of the reactions. A common means for representing the network structure underlying a system of chemical equations is that of the stoichiometry matrix. The stoichiometry matrix is an $n\times m$ matrix $\varvec{S}$, with each of the rows corresponding to a single species and each of the columns to a reaction. The matrix is populated such that its entries $s_{ij}$ give the net value of the stoichiometric coefficients (product minus reactant) of the *i*th species in the *j*th reaction. If the concentration of a particular species is not affected by a reaction, the corresponding entry is populated with a 0. Hence, the sign of the entry indicates whether the species is a net reactant or a net product in the relevant reaction. A positive sign implies that the species is a product (i.e. the number of molecules is increased by the reaction), whilst a negative sign indicates that the species is a reactant (i.e. the number of molecules is decreased). This matrix can be considered as mapping the vector of reaction rates $\varvec{v}(\varvec{x}(t),\varvec{p})$ to the change in species concentration. Hence it is possible to model the dynamics of the biochemical reaction network as a set of ODEs, such that

1 $$\begin{aligned} \dot{\varvec{x}}(t) = \varvec{S} \varvec{v}(\varvec{x}(t),\varvec{p}), \end{aligned}$$

where the over-dot represents the time derivative, such that $\dot{\varvec{x}}=\frac{\mathrm {d}\varvec{x}}{\mathrm {d}t}$. Systems of this type are typically solved as initial-value problems with some associated set of initial conditions, such that $\varvec{x}(0)=\varvec{x}_0$.

Whilst a description of dynamics in the form of Eq. (1) represents a useful way to understand a biochemical system, it is common to additionally explicitly account for input and output terms within a model. This can be achieved by employing a state-space representation of the form

2a $$\begin{aligned} \dot{\varvec{x}}(t)&= \varvec{f}(\varvec{x}(t),\varvec{p},\varvec{u}(t)), \end{aligned}$$

2b $$\begin{aligned} \varvec{y}(t)&= \varvec{g}(\varvec{x}(t),\varvec{p}), \end{aligned}$$

where $\varvec{u}(t)\in \mathbb {R}^l$ represents the set of model inputs and $\varvec{y}\in \mathbb {R}^p$ represents a set of model outputs. Hence, the dynamics of the state-variables are governed by the system of ODEs represented by Eq. (2a) and defined by the set of functions $\varvec{f}(\varvec{x}(t),\varvec{p},\varvec{u}(t))$. Additionally, the outputs are combinations of the original state-variables defined by some set of functions $\varvec{g}(\varvec{x}(t))$. This form can be related back to Eq. (1) by noting $\varvec{f}(\varvec{x}(t),\varvec{p},\varvec{u}(t))= \sum _{i=1}^m \varvec{s}_{ci}v_i(\varvec{x}(t) ),\varvec{p},\varvec{u}(t)),$ with $\varvec{s}_{ci}$ referring to the *i*th column of the stoichiometry matrix $\varvec{S}$.

The aim of model reduction is then to construct a simpler model in terms of a reduced set of state-variables $\tilde{\varvec{x}}\in \mathbb {R}^{\hat{n}}$ and parameters $\tilde{\varvec{p}}\in \mathbb {R}^{\hat{m}}$ such that either ${\hat{n}}<n$ or ${\hat{m}}<m$. A reduction in state-variables is justified on the principle that often trajectories in the phase space associated with a complex system of ODEs can be entirely contained within, or can be well approximated by, a lower-dimensional subspace. Finding the set of subspaces of a given dimensionality that best approximate the trajectories of interest is the primary goal of model reduction methods. Unfortunately, proving the optimality of a reduction for a given trajectory (or set of trajectories) is often not possible. Hence it is typical to seek an acceptable, as opposed to optimal, subspace to approximate the model. The construction of a reduced model within a given subspace is typically achieved via the Petrov–Galerkin projection.

Put simply, methods of model reduction can often be considered as a projection of the state-variables to a lower-dimensional subspace $\mathcal {V}:\; \text {dim}\left( \mathcal {V}\right) =\hat{n}$ of the original phase space, within which some relevant set of the system’s trajectories can be adequately approximated. Mathematically, it is the application of such a projection to obtain a reduced dynamical system that is underpinned by the Petrov–Galerkin projection (Antoulas 2005) as follows.

Assuming we have a given projection $\varvec{T}\in \mathbb {R}^{\hat{n}\times n}$ applied to create a reduced set of state-variables $\tilde{\varvec{x}}\in \mathbb {R}^{\hat{n}}$, such that

$$\begin{aligned} \tilde{\varvec{x}} = \varvec{T}\varvec{x}, \end{aligned}$$

and an associated generalised right-inverse $\bar{\varvec{T}}\in \mathbb {R}^{n\times \hat{n}}$, with $\varvec{T}\bar{\varvec{T}}=\varvec{I}_{\hat{n}}$, then the Petrov–Galerkin projection allows a reduced dynamical description of these state- variables as

$$\begin{aligned} \dot{\tilde{\varvec{x}}}&= \varvec{T}\varvec{S}\varvec{v}(\bar{\varvec{T}}\tilde{\varvec{x}}(t),\varvec{p}),\\ \bar{\varvec{y}}(t)&= \varvec{g}(\bar{\varvec{T}}\tilde{\varvec{x}}(t),\varvec{p}), \end{aligned}$$

when applied to a model of the form of Eq. (1). More details are given in Additional file 1—Supplementary information Section 1.1.

The quality of a given reduction is typically assessed by comparing some given metric of error $\varvec{\epsilon }$ between the output of the original and the reduced models, such that

3 $$\begin{aligned} \varvec{\epsilon } = \left\| \varvec{y}(t) - \bar{\varvec{y}}(t) \right\| . \end{aligned}$$

Methods of model reduction are therefore considered here as either a projection of the set of reactants or of the reactions to some subspace within which some subset of the original dynamical behaviour can be satisfactorily approximated as dictated by a given metric of error. Throughout this paper a range of methods from the literature will be introduced and outlined with reference to the general model forms represented by Eqs. (1) and (2). For several of the core methods we have also provided an example of more direct application to a nonlinear example model in Additional file 1—Supplementary information Section 2.

There are several issues that often arise with the reduction of large-scale biochemical models that in many ways define the suitability of model reduction methods in this context; these include nonlinearity, stiffness, high dimensionality, and the wide ranging aims of model reduction within this field.

*Nonlinearity* Systems of coupled, nonlinear ODEs are typically analytically intractable; hence, we are often constrained to using numerical approaches in the reduction of such models. Linearisation methods do exist for such systems, but their application typically incurs a relatively high degree of error which is often strongly dependent upon the parameterisation of the model and the nature of the nonlinearities seen.

*Stiffness* It is often the case in such models that reactions occur across a wide range of timescales. As a result the systems of ODEs governing these models are often considered numerically stiff and therefore require care when being simulated. In highly stiff systems some degree of numerical error under simulation is likely, even for specialised numerical methods, and can lead to issues for certain model reduction methods.

*High Dimensionality* Systems biology, due to its holistic approach, often produces very large systems of equations. Whilst model reduction obviously seeks to reduce such systems, this level of complexity has a number of associated issues. In particular such systems cannot be easily intuited, the numerical stability of reduction methods becomes especially important, and the computational calculation time for many methods in this setting can become prohibitive due to the combinatorial explosion in the range of possible model subspaces.

*Aims of Model Reduction* The choice of model reduction method employed is typically constrained by the aims of the researcher. For example, the optimal reduction that retains the biological meaning of the state-variables is likely to be non-optimal in a setting where transformations of the state-variables are permitted. The preferred reduction is also likely to differ if we select the reduction that can best approximate all state-variables as opposed to some subset, and depending upon the metric of error that is employed.

Note that whilst we have here outlined the process of modelling biochemical reaction networks in the context of the Law of Mass Action, most reduction methods reviewed in this paper are applicable in the broader context of general ODE systems. The Law of Mass Action typically represents the main theoretic basis for the deterministic modelling of systems biology type networks. However, it is also common that other terms such as Hill, logistic, or other mathematical functions are used to describe certain biological phenomena. Certain methods that are reviewed (e.g. Samal et al. 2015) do require that the model contains only polynomial terms or, in certain instances, that the model has a specific structure (e.g. Löwe et al. 2016) or that it is linear (e.g. Sunnåker et al. 2010). Where the methods do require a more specific structure than a general system of ODEs, this will be highlighted as part of the review.

## Model Simplification Methods

Conservation analysis, nondimensionalisation, and model decomposition are three commonly applied techniques for the simplification and analysis of models of biochemical systems. All three methods are strongly related to model reduction techniques and can be seen as simplifying the representation of a model without incurring any associated error cost. Hence they can be considered to produce simplified model realisations as opposed to acting as true model reductions. These techniques are often applied prior to the use of model reduction methods with the aim of obtaining the simplest or most easily manipulated version of a system.

### Conservation Analysis

Models of biochemical reaction networks commonly possess subsets of reactants that, under a given linear combination, remain constant at all times (Klipp et al. 2013). These subsets are typically referred to as conserved moieties and the specific linear combinations as conservation relations. In combination with the system of ODEs described by Eq. (2a), the existence of conservation relations implies that the model can be expressed as a system of differential algebraic equations (DAEs), such that

4a $$\begin{aligned} \dot{\varvec{x}}(t)&= \varvec{f}(\varvec{x}(t),\varvec{p},\varvec{u}(t)), \end{aligned}$$

4b $$\begin{aligned} 0&= \varvec{{\varGamma }} \varvec{f}(\varvec{x}(t),\varvec{p},\varvec{u}(t)), \end{aligned}$$

where $\varvec{{\varGamma }}$ is an $h\times n$ matrix referred to as the conservation matrix, the rows of which represent the linear combinations of reactants that are constant in time. As Eq. (4b) is linear following integration, it can be solved explicitly and used to eliminate up to *h* state-variables and their associated ODEs from the system defined by Eq. (4a). This replacement of state-variables via the algebraic exploitation of conservation relations is a common first step in the analysis of biochemical reaction networks and, for large systems, typically results in a reduction of 10–15% of the state-variables (Vallabhajosyula and Sauro 2006).

For small networks conservation relations are usually obvious and easily exploited. For very large systems, however, these relations are often not readily apparent. As such it is common to turn to algorithmic approaches for finding the conservation matrix $\varvec{{\varGamma }}$. As is discussed in Reder (1988), this can be achieved by computing the left null-space (and hence the linear dependencies) of the network’s associated stoichiometry matrix. A review of a range of methods to find the left null-space of this matrix, including Gaussian elimination and singular value decomposition, can be found in Sauro and Ingalls (2004). Such methods, however, are often numerically unstable for systems of very high dimension, which can lead to some conservation relations being missed. A more numerically stable method based upon the construction of a QR decomposition via Householder reflections has been developed by Vallabhajosyula et al. (2006). An example of the application of algorithmic conservation analysis to a nonlinear example model is provided in Additional file 1—Supplementary information Section 2.1.

### Nondimensionalisation

Nondimensionalisation refers to a process of scaling the variables in a system such that the physical units are removed from the model (Murray 2002). In the case of the systems considered here, this is most often units of molecular concentration and time. There are number of purposes for nondimensionalisation in the analysis of biochemical systems—primary amongst these is its use in accessing characteristic or intrinsic differences in scale between the components of the reaction network. Usually these are represented by ratios of rate parameters and conserved values that enable greater intuition into how the parameterisation of a model governs its behaviour. As will be discussed in later sections, these characteristic parameters can be crucial for the application of model reduction methods based upon singular perturbation theory.

For a model represented in the stoichiometric form given by Eq. (1) the aim of nondimensionalisation is to rescale the state-variables $x_i(t)\in \varvec{x}(t)$ and the independent variable *t* such that they are dimensionless. This produces a transformation to rescaled variables of the form

5a $$\begin{aligned} x_i \rightarrow \hat{x}_i: \, x_i =\,&\, a_i\hat{x}_i, \end{aligned}$$

5b $$\begin{aligned} t \rightarrow \tau :\,t =\,&\, b\tau , \end{aligned}$$

where $a_i\in \varvec{a}$ each typically represent some, to be determined, constant of molecular concentration and *b* represents a constant of time. Note that the transformation of all state-variables given by Eq. (5a) can hence be written as $\varvec{x}=\varvec{{\varTheta }}\hat{\varvec{x}}$ where $\varvec{{\varTheta }}$ is an $n\times n$ diagonal matrix of the form $\varvec{{\varTheta }} = \text {diag}\left( a_1,\ldots , a_n\right)$. Therefore, applying this transformation to the original system (1) yields a nondimensionalised system of the form

6 $$\begin{aligned} \frac{\mathrm {d}\hat{\varvec{x}}(\tau )}{\mathrm {d}\tau } = b\varvec{{\varTheta }}^{-1}\varvec{S}\varvec{v}\left( \varvec{{\varTheta }}\hat{\varvec{x}}(\tau ),\hat{\varvec{p}}\right) . \end{aligned}$$

This yields a nondimensionalised parameter set $\tilde{\varvec{p}}$ with entries representing specific ratios of the original parameters $\varvec{p}$. Often, this approach can result in a reduction in the dimension of the new parameter set $\tilde{\varvec{p}}$ by finding ratios that are fixed to one irrespective of the original parameterisation. This does not, however, result in a reduction in the number of modelled reactions and hence does not reduce model complexity as previously defined. Additionally, the dimensionless parameters may lose their innate biological meaning as the ratios they represent may not always hold particular biological significance.

### Model Decomposition

Biochemical reaction networks are often highly modular in nature (Hartwell et al. 1999; Milo et al. 2002; Bruggeman et al. 2002; Sauro 2008). This implies that the elements (species or reactions) of most networks in this context, as compared to a randomly generated network, can be more easily partitioned into sub-networks that are highly connected within themselves and possess a low number of connections to elements outside of their partition. Additionally, complex phenomenological behaviours can often be shown to be driven by small sub-networks contained within the larger network (Lauffenburger 2000; Tyson et al. 2003). The approach of dividing the system into interacting sub-networks (often referred to as modules) is known as model decomposition. Given the high degree of network modularity common in this field and the likelihood of certain modules to dominate the dynamical behaviour of interest, model decomposition is an attractive technique in the modelling of biochemical systems.

Methods of model decomposition are also highly complementary to methods of model reduction as they can be used to separate the system into modules of differing ‘importance’ and hence be used to guide reduction. For example, it may be the case that only those portions of a signalling pathway model addressing the initial receptor binding of an extracellular ligand and the phosphorylation of a particular protein downstream are of interest to the modeller. In this instance it may make sense to decompose the system into two modules representing these portions and a third module describing the ‘unimportant’ components of the network. This can then be used to guide model reduction such that the module deemed unimportant can be reduced in isolation and, potentially, approximated with a lower degree of accuracy than the important modules.

**Fig. 1:**
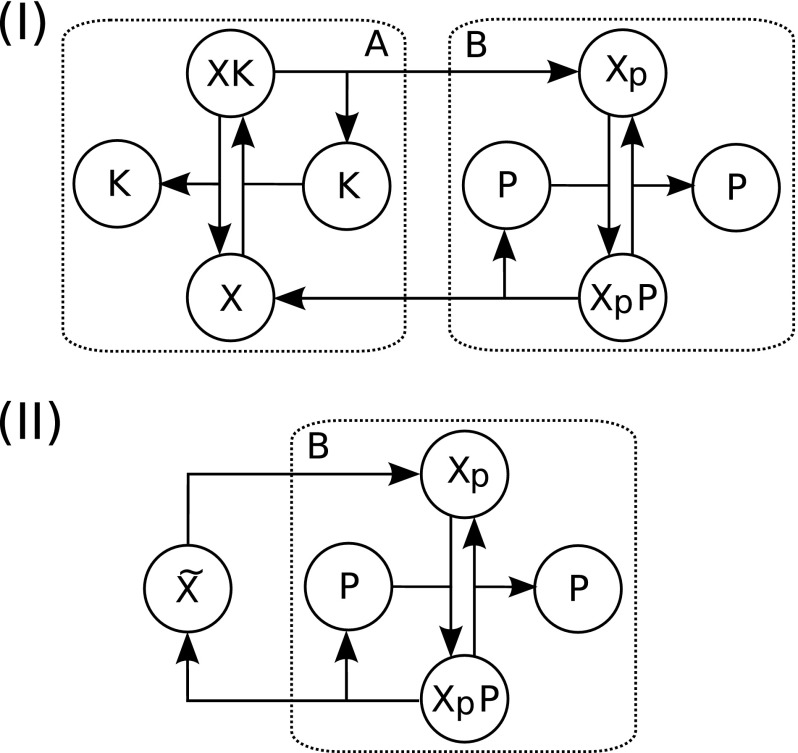
Schematic depiction of a simple phosphorylation cycle and a potential decomposition of the network. **I** The network depicted here represents a simple enzymatic phosphorylation cycle—a kinase *K* mediates the phosphorylation of a protein *X*, whilst a phosphatase *P* performs the process of dephosphorylation. Here a biologically guided decomposition of the network into two sub-modules *A* and *B* is depicted—with *A* representing the unphosphorylated protein and the kinase binding step, *B* representing the phosphorylated protein and the phosphatase binding step, and only the phosphorylation and dephosphorylation reactions linking the two sub-modules. **II** An example of a decomposition guided model reduction of the phosphorylation cycle. In this example module *A* representing the kinase binding has been reduced to a single state-variable, whilst the full biological detail of the phosphatase binding and dephosphorylation of *X* has been retained

As an example, consider the phosphorylation cycle [a description of phosphorylation cycles and their modelling can be found in Salazar and Höfer (2009)] depicted in Fig. 1a. Given a system of this form a biologically reasonable decomposition is to partition the system into phosphorylation and dephosphorylation modules as depicted in Fig. 1 as modules *A* and *B*, respectively. If, for example, the modeller was primarily interested in the dephosphorylation module, it might be possible to reduce the phosphorylation module significantly, as shown in Fig. 1b, whilst still retaining an accurate description of the biological mechanisms of interest.

A full review of decomposition methods is beyond the scope of this paper. A wide range of approaches for finding suitable decompositions can be found in the literature (Holme et al. 2003; Saez-Rodriguez et al. 2004, 2005; Vecchio and Sontag 2009; Kaltenbach et al. 2011; Anderson et al. 2011; Sivakumar and Hespanha 2013; Prescott and Papachristodoulou 2014). Related methods for determining whether a given model can be found as a sub-network in a larger system have also been discussed (Gay et al. 2010). Sun and Medvedovic (2016) have proposed the decomposition of models into linear and nonlinear sub-modules for the purpose of parameter fitting via Rao–Blackwellised particle filters decomposition methods. Additionally, approaches for determining which sub-modules of a network drive particular dynamical behaviour of a model (oscillations, for example) (Schmidt and Jacobsen 2004) may have a particular applicability within the context of model reduction, guiding the use of reduction so as to preserve phenomena of interest.

## Model Reduction Methods

### Timescale Exploitation Methods

**Fig. 2:**
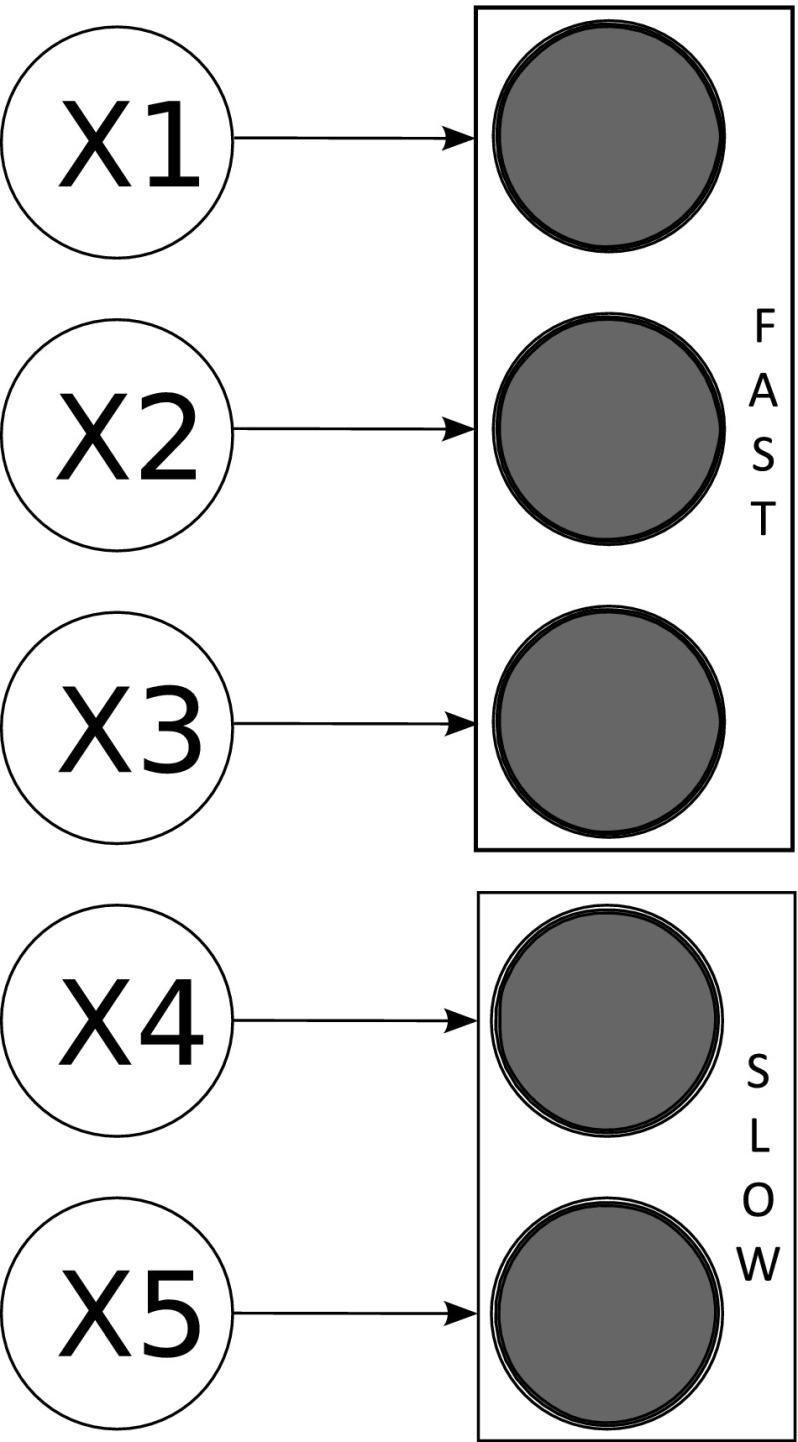
A schematic depiction of model reduction via timescale decomposition. Here state-variables are either grouped as slow or fast. This allows each group to be excluded via approximation at differing timescales of interest. For example, for dynamics at fast timescales it may be reasonable to assume the slow variables are constant, hence producing a reduction in state-variables

Timescale exploitation methods are the most commonly applied approaches for reducing models of biochemical systems. Methods in this area seek to partition the system into different timescales by exploiting the often wide variation (commonly spanning orders of magnitude) between individual reaction rates and the speed with which the differing reactants equilibrate. Such variation is common within biochemical reaction networks. Differences in timescales can, for instance, allow some reaction processes to be classed as fast or slow relative to the dynamics of interest. These differences can be exploited to reduce a model; for example, relatively slow dynamical processes can be assumed to be constant or relatively fast processes to equilibrate rapidly on the timescale of interest. Figure 2 provides a schematic depiction of the concept of dividing species into fast and slow timescales for the purpose of model reduction.

Given this definition, timescale exploitation methods fall into two major groups: those that preserve the meaning of the state-variables in the dynamical description of the system (coordinate preserving) and those that do not (coordinate transforming). Based upon these classifications, the following sections provide an overview of the most commonly applied methods and recent publications addressing their application in the context of biochemical reaction networks.

#### Coordinate Preserving Timescale Methods

These methods are based upon identifying either species or reactions which can be considered as exhibiting ‘fast’ dynamics in comparison with the remainder of the network, hence partitioning the system into fast and slow components. Often this involves finding some nondimensionalisation that exposes a small parameter $\delta \ll 1$ that can be used to distinguish between species and reactions occurring on fast and slow timescales. Once such a representation has been found, application of singular perturbation theory enables the reduction of the system.

Singular perturbation for the reduction of systems of first-order ODEs was originally developed by Tikhonov (1952). His original paper is in Russian, but an excellent synopsis in English is given by Klonowski (1983) which guides the description provided here.

Tikhonov’s theorem on dynamical system states that, under certain conditions, if a system of first-order differential equations can be expressed in the form

7a $$\begin{aligned} \dot{\varvec{x}}_1(t)&= \varvec{f}\left( \varvec{x}_1,\varvec{x}_2,t\right) , \end{aligned}$$

7b $$\begin{aligned} \delta \dot{\varvec{x}}_2(t)&= \varvec{g}\left( \varvec{x}_1, \varvec{x}_2,t\right) , \end{aligned}$$

where Eq. (7a) is commonly referred to as the degenerate system and (7b) as the adjoined system, then as $\delta \rightarrow 0$ the solution of the whole system tends to that of the degenerate system, such that

8a $$\begin{aligned} \dot{\varvec{x}}_1(t)&= \varvec{f}\left( \varvec{x}_1, \varvec{x}_2,t\right) , \end{aligned}$$

8b $$\begin{aligned} \varvec{x}_2 (t)&= \varvec{\phi }\left( \varvec{x}_1,t\right) , \end{aligned}$$

with $\varvec{\phi }\left( \varvec{x}_1,t\right)$ a root of the equations $\varvec{g}\left( \varvec{x}_1, \varvec{x}_2,t\right) =0$. Clearly, Eq. (8b) can be substituted into Eq. (8a) to produce a reduced system of ODEs in terms only of state-variables $\varvec{x}_1(t)$.

In order for this reduction to hold, Tikhonov’s theorem requires the following conditions to be met:

1. $\varvec{x}_2(t) = \varvec{\phi }\left( \varvec{x}_1,t\right)$ must be an ‘isolated’ (i.e. non-repeated) root of the equations $\varvec{g}\left( \varvec{x}_1, \varvec{x}_2,t\right) =0$;
2. the solution $\varvec{x}_2(t) = \varvec{\phi }\left( \varvec{x}_1,t\right)$ must be a stable steady state of the adjoined system (7b); and
3. the initial conditions used in the reduced system must be in the basin of attraction for this steady state of the adjoined system.

This approach to reduction is commonly referred to as singular perturbation. Assuming $\delta =0$ is equivalent to a first-order truncation of the asymptotic expansion in terms of $\delta$. Higher-order approximations can often be computed, potentially providing more accurate reduced models for somewhat larger values of $\delta$. Kokotovic (1984) additionally demonstrates how singular perturbation can be applied to a control-theoretic state-space model in the form of (2).

*Species Partitioning* In the case where a timescale separation for the rates of species evolution can be observed, it is possible to partition $\varvec{x}$ such that

9 $$\begin{aligned} \varvec{x}(t) = \left( \begin{array}{c} \varvec{x_s}(t) \\ \varvec{x_f}(t) \end{array}\right) , \end{aligned}$$

where $\varvec{x_s}(t)$ represents those state-variables that evolve slowly in comparison with $\varvec{x_f}(t)$. For such a partitioning of a system to exist, it must be possible, via some nondimensionalisation, to express it in the form

10 $$\begin{aligned} \left( \begin{array}{c} \dot{\varvec{x}}_{\varvec{s}}(t) \\ \delta \dot{\varvec{x}}_{\varvec{f}}(t) \end{array} \right) = \left( \begin{array}{c} \varvec{S}_s \\ \varvec{S}_f \end{array}\right) \varvec{v}\left( \varvec{x_s}(t), \varvec{x_f}(t),\varvec{p}\right) , \end{aligned}$$

with the positive constant $\delta \ll 1$ corresponding to the difference in evolution speeds for the different species. Setting $\delta \dot{\varvec{x}}_{\varvec{f}}(t) \approx 0$ yields the system of differential algebraic equations (DAEs)

11a $$\begin{aligned} \dot{\varvec{x}}_{\varvec{s}}(t)&= \varvec{S}_s \varvec{v}\left( \varvec{x_s}(t), \varvec{x_f}(t),\varvec{p}\right) , \end{aligned}$$

11b $$\begin{aligned} 0&= \varvec{S}_f \varvec{v}\left( \varvec{x_s}(t), \varvec{x_f}(t),\varvec{p}\right) . \end{aligned}$$

Clearly, where Eq. (11b) can be solved, variables $\varvec{x_f}(t)$ can be eliminated from Eq. (11a) to yield a reduced model. This method of model reduction is commonly referred to as the quasi-steady-state approximation (QSSA), and its most famous application is in reducing the Michaelis–Menten equation as outlined by Briggs and Haldane (1925). An example of the direct application of the QSSA to a nonlinear example model can be found in Additional file 1—Supplementary information Section 2.3.

Such a reduction is valid where the timescale of the slowest fast species (${\tau }_{f,\text {max}}$) is significantly shorter than the timescale of the fastest slow species (${\tau }_{s,\text {min}}$), such that ${\tau }_{f,\text {max}}\ll {\tau }_{s,\text {min}}$. This is guaranteed to be the case where a formulation for the model of the form (10) can be found with $\delta \ll 1$; typically such a formulation is found via searching through possible nondimensionalisations of the system.

Petrov et al. (2007), for example, recently applied the QSSA to a nondimensionalised and singularly perturbed model of the extracellular regulatory kinase (ERK) signalling pathway regulated by a Raf kinase inhibitor protein (RKIP). They showed that an 11-dimensional system can be reduced to 5 dimensions, and crucially, this reduced model can, unlike the original system, be solved analytically. This enables the biological insight that the RKIP protein only provides a regulatory role in the ERK pathway far from the system’s steady state.

A number of variations of the QSSA approach can also be found in the literature; Schneider and Wilhelm (2000) discussed how the QSSA can be extended to singular, singularly perturbed systems and how this approximation can be extended to higher orders via asymptotic expansion. Vejchodskỳ et al. (2014) and Vejchodskỳ (2013) have introduced the delay quasi-steady- state approximation (DQSSA), enabling the QSSA method to compensate for the time error incurred by forcing the approximation that the timescale of the fast species is equal to zero. This time error can be particularly problematic for oscillatory systems where it can result in a mismatched phase. Compensating for this effect can greatly increase the accuracy of the QSSA in the case of such systems. Their approach is demonstrated via application to a 9-dimensional model of circadian rhythms which can be reduced to 2 dimensions; the standard QSSA incurs a 30% error for this reduction due to a mismatch in phase, whereas the DQSSA only incurs a 2% error.

Unfortunately, the QSSA is somewhat limited in the models it can be applied to, as it requires that the species exhibit a clear separation in timescales and a formulation amenable to singular perturbation. For simpler examples, searching through the range of possible nondimensionalisations and employing intuition of the system in order to find such a formulation is often feasible. For very large models, however, such an approach can be prohibitive due to the combinatorial explosion in the range of possible model representations. As a result of the difficulties that commonly occur in finding a suitable partitioning of species, a number of publications in this area are dedicated to providing algorithmic methods for determining species that can potentially be considered ‘fast’. Choi et al. (2008), for example, have devised an algorithmic approach to rank the timescale factors of species via analysis of the system’s Jacobian after a short initial transient period. Similarly, West et al. (2014) have recently introduced a notion of ‘speed coefficients’ that can be calculated for the state-variables of a model via analysis of the system’s Jacobian and used to guide the fast/slow partitioning of the species.

The zero-derivative principle (ZDP) provides a computational approach for extending the QSSA to higher-order approximations (see Additional file 1—Supplementary information Section 1.2). Härdin et al. (2009) have demonstrated use of the ZDP for the reduction of biochemical reaction networks via application to the Michaelis–Menten enzyme–substrate model and a phosphotransferase system (PTS) within the context of glucose transport. In the case of the PTS model it was demonstrated that a first-order ZDP approximation enabled the reduction of the original 9-dimensional system to a single state-variable whilst retaining a high degree of accuracy which was not attainable solely under the QSSA.

*Reaction Partitioning* An alternative approach to partitioning the species $\varvec{x}(t)$ is to instead partition the reaction rates $\varvec{v}\left( \varvec{x}(t), \varvec{p}\right)$ into fast and slow groups, such that

12 $$\begin{aligned} \varvec{v}\left( \varvec{x }(t), \varvec{p}\right) = \left( \begin{array}{c} \varvec{v_s}\left( \varvec{x }(t), \varvec{p}\right) \\ \delta ^{-1}\varvec{v_f}\left( \varvec{x}(t), \varvec{p}\right) \end{array}\right) , \end{aligned}$$

with $\delta \ll 1$. Here $\varvec{v_s}\left( \varvec{x }(t), \varvec{p}\right)$ corresponds to the slow reaction rates and $\varvec{v_f}\left( \varvec{x}(t), \varvec{p}\right)$ to those that can be considered fast in comparison (as denoted by the associated small parameter $\delta$). This leads to a dynamical system of the form

13 $$\begin{aligned} \dot{\varvec{x}}(t) = \left( \varvec{S}_s \,\, \varvec{S}_f\right) \left( \begin{array}{c} \varvec{v_s}\left( \varvec{x}(t), \varvec{p}\right) \\ \delta ^{-1}\varvec{v_f}\left( \varvec{x}(t), \varvec{p}\right) \end{array}\right) , \end{aligned}$$

where $\varvec{S}_s$ and $\varvec{S}_f$ represent submatrices of the stoichiometry matrix comprising those columns corresponding to the slow and fast reactions, respectively.

Hence, the dynamics for the species concentrations $\dot{\varvec{x}}(t)$ can be decomposed into fast and slow contributions as a sum, such that $\dot{\varvec{x}}(t) = \left[ \dot{\varvec{x}}(t)\right] _{\varvec{s}} + \left[ \dot{\varvec{x}}(t)\right] _{\varvec{f}}$. Note here that, unlike the equivalent terms in the species partitioning case, $\left[ \dot{\varvec{x}}(t)\right] _{\varvec{s}}$ does not necessarily correspond to a proper subset of $\varvec{x}(t)$—rather it represents the slow dynamical contribution of each reaction to all of the modelled species concentrations.

Taking the approximation $\delta \rightarrow 0$, singular perturbation yields

14a $$\begin{aligned} \left[ \dot{\varvec{x}}(t)\right] _{\varvec{s}}&= \varvec{S}_s \varvec{v_s}\left( \varvec{x}(t), \varvec{p}\right) , \end{aligned}$$

14b $$\begin{aligned} 0&= \varvec{S}_f \varvec{v_f}\left( \varvec{x}(t), \varvec{p}\right) . \end{aligned}$$

As $\varvec{x}(t)$ still depends on both the slow and fast dynamical contributions, the aim is to solve Eq. (14b) in such a way that (14a) can be decoupled from the fast contributions, leaving a reduced model that accurately describes the slow timescale. This method operates under the assumption that certain reactions occur fast enough so as to be approximated as equilibrating instantaneously; hence, it is commonly referred to as the rapid equilibrium approximation (REA). The most famous application of the REA is Michaelis and Menten’s original reduction of the enzyme–substrate reaction model (Michaelis and Menten 1913).

The rapid equilibrium approximation has been applied in the work of Vora and Daoutidis (2001), Gerdtzen et al. (2002) and Gerdtzen et al. (2004) to a number of models, in particular a model of the glycolytic pathway in *Saccharomyces cerevisiae* where they were able to reduce the system from 21 to 18 reactions whilst maintaining a high degree of accuracy, and a model of central carbon metabolism in humans where they were able to similarly achieve a reduction from 25 to 20 reactions.

More recently, Prescott and Papachristodoulou have developed a variant of this approach (Prescott and Papachristodoulou 2013, 2014) that further generalises the process of dividing such systems based upon differences in reaction timescales and hence partitioning the columns of the stoichiometry matrix. This work yielded an automatable model decomposition method they term layering (Prescott and Papachristodoulou 2014). They highlight the fact that such an approach can present a more natural means of model decomposition as opposed to the traditional approach of partitioning species into modules.

*Finding Timescale Partitions* The main difficulty associated with these timescale partitioning methods is that of finding a formulation of the system for which an appropriate parameter $\delta \ll 1$ can be identified. A range of approaches addressing this issue have been discussed in the literature.

Noel et al. (2012, 2013), Soliman et al. (2014) and Radulescu et al. (2015) have proposed, developed and refined an approach of model tropicalisation for the reduction of biochemical models—this is a method of model abstraction which can guide the application of both the species- and reaction-based singular perturbation approaches described above. Samal et al. (2015) further develop the method of tropicalisation in the context of systems with entirely polynomial governing equations by introducing an algorithm allowing the automatic computation of tropical equilibrations based upon the Newton polytope and edge filtering.

Holland et al. (2011) have also provided an a posteriori means of analysing systems for the existence of possible QSSA or REA simplifications. The system is simulated under two conditions—the introduction and the removal of a fixed input into the system. The trajectories of these simulations are then plotted in each of the 2-dimensional phase planes between all possible pairs of state-variables. In each case the hysteresis between these two trajectories is used to judge the possibility that each pair can be considered to rapidly equilibrate with respect to one another and hence guide application of the timescale exploitation methods described throughout this section. This method was applied to a 25-dimensional model of $\beta _1$-adrenergic signalling, where it was shown that a 6-dimensional reduced model was capable of accurately capturing the original system’s dynamics.

Löwe et al. (2016) demonstrate that for models which can be recast in the form of S-systems, it is always possible to algorithmically rank the timescales of species and to obtain a simple description of how this varies with model parameterisation. This is achieved by expressing the system in the form of a generalised Lotka–Volterra model through the analysis of a specific constant matrix and application of singular value decomposition, and it is then possible to study how the timescales of the state-variables depend upon both the specific parameterisation and stoichiometry of the system. This approach is demonstrated via application to three real-world examples a model of yeast glycolysis, the citric acid (TCA) cycle and purine metabolism.

#### Coordinate Transforming Timescale Methods

In the previous section it was discussed that often a nondimensionalisation of a system was required in order to clearly expose the timescale differences between species and reactions. In this section, however, it is shown that a change of basis for the state-variables can often be used to obtain a transformed model where timescale separation is significantly more readily apparent and exploitable. Such approaches can often lead to lower-dimensional and more accurate model reductions than the methods so far discussed. However, this is weighed against the fact that the transformations employed will often obfuscate the biological interpretability of the reduced dynamical system.

The methods outlined in this section aim to find a transformation of the state-variables under which the fast and slow dynamics can be decoupled and then used to reduce the system whilst retaining a high degree of accuracy between the simplified and original models. In essence, such methods seek a low-dimensional manifold within the phase space of the system upon which trajectories of interest for the dynamical model can be satisfactorily approximated on the timescale of interest.

Usually the aim is to describe the dynamics on the slow timescales and thus seek a manifold that can approximate trajectories after a short initial transient period through to steady-state. This is commonly known as an inertial manifold (or in special cases, as the slow manifold Debussche and Temam 1991). The methods discussed in this section provide approximations of such manifolds.

The simplest example involves linearisation and transformation of the state-variables into the system’s eigenbasis. First note that a system of the form described by Eq. (1) can be linearised (i.e. approximated by a linear system of ODEs) around a given state $\varvec{x}_c$ of the system by calculating the Jacobian matrix

15 $$\begin{aligned} \varvec{J}_{\varvec{x}_c}= \left. \varvec{S}\varvec{E} \right| _{\varvec{x}(t) = \varvec{x}_c}, \end{aligned}$$

with $\varvec{E}$ commonly referred to as the elasticity matrix, whose entries are given by

16 $$\begin{aligned} \varvec{E} = \left\{ e_{ij}= \frac{\partial v_i\left( \varvec{x}, \varvec{p}\right) }{\partial x_j}\right\} . \end{aligned}$$

Then, via a first-order Taylor expansion, the system can be approximated in the neighbourhood of $\varvec{x}_c$ by

17 $$\begin{aligned} \dot{\varvec{x}}(t) \approx \varvec{S}\varvec{v}\left( \varvec{x}_c, \varvec{p}\right) + \varvec{J}_{\varvec{x}_c}\left( \varvec{x}(t) - \varvec{x}_c\right) . \end{aligned}$$

The eigenvectors $\nu _i$, for $i=1,\ldots , n$, of $\varvec{J}_{\varvec{x}_c}$ represent directions of movement around this point in phase space, and the corresponding eigenvalues $\lambda _i$ determine the speed of movement along that direction. Hence if the state-variables are transformed so as to correspond with the directions of the eigenvectors (i.e. into the eigenbasis), clear timescales $\tau _i=-1/\left| {\text {Re}}(\lambda _i)\right|$ can be associated with each new variable. If there is a sufficiently large gap between any two successive eigenvalues (i.e. an eigengap), a timescale decomposition of the transformed state-variables into slow and fast groups is possible, and hence, singular perturbation can be applied to obtain a reduced system. Unfortunately, if some of the eigenvalues are tightly clustered or are replicated, standard eigendecomposition approaches may suffer issues of numerical inaccuracy.

The intrinsic low-dimensional manifold method (ILDM), originally developed as a means of model reduction by Maas and Pope (1992) within the context of combustion chemistry, provides a numerically stable means of applying an eigenbasis decomposition. ILDM has seen a number of applications within the field of biochemical modelling, and a more detailed account of the methodology is given in Additional file 1—Supplementary information Section 1.3. Vallabhajosyula and Sauro have also provided a brief review of the ILDM method within the context of biochemical reaction networks (Vallabhajosyula and Sauro 2006). An example of the direct application of the ILDM method to a nonlinear example model can also be found in Additional file 1—Supplementary information Section 2.4.

Notably, Zobeley et al. (2005) have developed a time-varying form of the ILDM method where the time course of the model is split into multiple intervals with differing reductions. This approach was demonstrated via application to a model of peroxidase–oxidase reaction coupled with enzyme activity consisting of 10 ODEs. Under their approach the model could be reduced to between 3 and 5 state-variables at each time-interval whilst maintaining a high degree of accuracy. Surovtsova and Zobeleya (2006) have also examined this approach via application to a model of glycolysis in yeast cells. In particular they sought to answer the question of how far the ILDM continues to provide an accurate timescale decomposition away from the point of linearisation $\varvec{x}_c$.

Surovtsova et al. (2009) have developed a highly automatable and time-dependent form of the ILDM method for implementation in the COPASI software package (Hoops et al. 2006). Time dependency is achieved by not decoupling the fast and slow transformed state-variables found under the ILDM. Here, instead, the QSSA is applied to the species that are shown to contribute most to the set of fast transformed state-variables. Hence, although it has its roots in ILDM, this approach is coordinate preserving as opposed to employing a change of basis. This approach is demonstrated via application to models of calcium oscillation and glycolysis in *Saccharomyces cerevisiae*. In both cases good reductions could be obtained, with a maximal relative error of around 0.5% across all reactants in the glycolysis case.

Bykov and Goldshtein (2016) outline a similar method to the ILDM termed the global quasi-linearisation method (GQL) that can be used to exploit fast/slow decompositions of the system. By combining the conservation relations and the singularly perturbed eigendecomposition of the systems GQL matrix, it is possible to replace a number of species with algebraic relations and hence reduce the system. This approach is demonstrated for a 28-dimensional system describing the intracellular signalling of FAS induced apoptosis; this system was reduced to 15 dimensions whilst incurring <1% relative error.

An alternative coordinate transforming method based upon timescale decomposition is that of computational singular perturbation (CSP). The CSP method was originally published in 1985 by Lam (1985) and further developed in a series of papers by Lam and Goussis (1991), Lam (1993) and Lam and Goussis (1994). More recent work by Kaper and Kaper (2002) and Zagaris et al. (2004a, b) has provided a rigorous analysis of the asymptotic behaviour of CSP and its relationship to other timescale-based methods such as ILDM.

Like ILDM, CSP seeks to provide a general framework for applying a timescale decomposition where no obvious nondimensionalisation exposing a singularly perturbed form can be found. This is again achieved by applying a change of basis. Unlike the ILDM method, however, CSP seeks to transform the set of reactions into a new basis that exposes clear timescale differences between the set of transformed reactions. The fast transformed reactions can then be assumed to equilibrate instantaneously, and hence their dynamical contribution can be neglected in a reduced model.

In computing these transformed reaction rates, CSP also yields timescale estimates for the original set of reactions and state-variables. These timescale indices can be to guide the application of more traditional methods of reduction such as QSSA or REA. CSP is a highly automated approach that iteratively constructs a change of basis for the reactions. In doing so, application of CSP can provide significant analytical insight into the driving factors of a dynamical system. Further details on the application of CSP can be found in Additional file 1—Supplementary information Section 1.4.

Surovtsova et al. (2012) have discussed the implementation the CSP algorithm in the COPASI software package and also demonstrated its application for the reduction of a model of glycolysis in *S cerevisiae*. Specifically, they showed the use of the method in guiding the application of the QSSA and the REA. They were hence able to reduce the original system, involving 22 state-variables and 24 reactions, to a 17-dimensional model detailing 19 reactions that remained accurate for a wide range of dynamical regimes.

Kourdis et al. (2008, 2010) similarly applied the method to a model of glycolysis in *S. cerevisiae*. Here, however, they were only concerned with the long-term dynamical description of the system on a limit-cycle and, additionally, the transformation of the reactions into a new basis was permitted. Under this approach they were able to demonstrate that the limit-cycle contained within an 11- dimensional manifold and that evolution along this trajectory could be accurately described using only three state-variables. The publication also explored the use of CSP in guiding conventional model reduction approaches, but found that a 10-dimensional reduction attained via guided application of QSSA and REA performed significantly worse than that obtained via the construction of a transformed reaction basis. In a further work, Kourdis et al. (2013) sought to analyse a model of the NF-$\kappa$B signalling system via application of CSP and the computation of timescale indices, but did not propose a specific reduced model.

#### Summary of Timescale Exploitation Methods

Coordinate transforming model reduction methods can often be applied with good results to models for which an exploitable, singularly perturbed form is not readily available. Additionally, these methods are often algorithmic, automatable and readily applicable to very large systems of ODEs. As a result of these advantages, coordinate transforming timescale-based methods can often produce lower- dimensional and more accurate reduced models than coordinate preserving alternatives.

Unfortunately, coordinate transformations can sometimes undermine the purpose of seeking a model reduction. In particular the reduced biochemical network will often lose some degree of biological intuitiveness as the transformed state-variables can only be interpreted as combinations of the originals. Whilst it is possible to map reduced state-variables back to the original ones, the network structure of the reduced model will typically be biologically inscrutable. Hence the choice of timescale exploitation method must be carefully considered along with the intended aim of the reduction.

### Optimisation-Based Methods and Sensitivity Analysis

Optimisation methods seek to maximise or minimise a function within a given range of acceptable perturbations. Such approaches have broad applicability within the context of model reduction; this can be considered as an optimisation problem, where the number of dimensions is defined as an objective function that reduction seeks to minimise subject to the constraint that the error $\epsilon$ (from Eq. 3) remains sufficiently small.

To obtain an optimal solution to such a problem it is common to take one of two approaches:

1. either seek to measure how ‘sensitive’ the constraint variable $\epsilon$ is to changes in the network’s structure or parameterisation and use this knowledge to guide a reduction. These methods are referred to here as sensitivity analysis-based approaches; or
2. employ a trial-and-error-based approach where multiple reduced systems are tested. From the range of possibilities, an optimal or near-optimal reduced system is returned. These methods are referred to as optimisation-based approaches.

A brief overview of each approach is provided here.

#### Sensitivity Analysis

Sensitivity analysis can be local or global and represents a commonly applied methodology in the systems biology literature (Zi 2011). It is typically employed to determine how robust the system’s response is to fluctuations in parameter values; however, sensitivity analysis can also be used in model reduction to guide the elimination of the least influential reactions or species in a system.

**Fig. 3:**
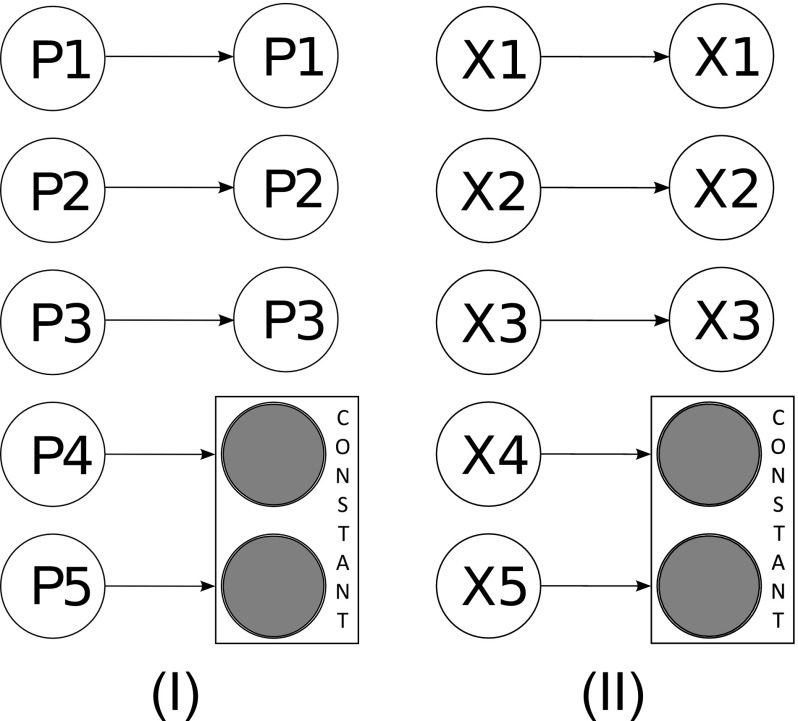
Schematic depiction of sensitivity analysis versus optimisation. **I** Sensitivity analysis allows the ranking of the relative importance of the parameters on the outputs of interest. The least influential parameters can be fixed as constant lessening the burden of parameter fitting or can enable model reduction through the elimination of associated parameters. **II** The optimisation approaches differ in that they typically aim to eliminate the least influential state-variables by fixing them to be constant in time

Given the state-space representation of Eq. (2), the aim of sensitivity analysis is to determine how the output $\varvec{y}(t)$ changes under perturbations to the parameters $\varvec{p}$ and the state-variables $\varvec{x}(t)$. To then reduce the system, the most common approach is simply to eliminate those species or parameters found to be the least sensitive in affecting the model. This is typically achieved by setting insensitive parameters equal to zero and fixing insensitive state-variables to some constant value (typically its steady-state value). Figure 3 provides a schematic depiction of this approach to model reduction. Note that this method of sensitivity analysis preserves the meaning of the reduced state-variables and reactions as no transformation is employed.

*Local Sensitivity Analysis* Local sensitivity analysis studies the response of the system to small perturbations in the model parameterisation around some specified operating point $\varvec{p} = \varvec{p}^*$. More specifically, such an analysis usually aims to describe variation of the model’s state-variables with respect to parameter variation by constructing a sensitivity matrix $R(t) = \left\{ r_{ij}(t)\right\}$ where the entries represent the effect of perturbing the *j*th model parameter on the *i*th state-variable. As is discussed in Kirch et al. (2016), for example, it is also common to normalise these indices of sensitivity such that measures of sensitivity remain invariant under the rescaling of state-variables. Further details on computing the sensitivity matrix are provided in Additional file 1—Supplementary information Section 1.5, and an example of the direct application of normalised, local sensitivity analysis to a nonlinear example model can be found in Section 1.5. Once a matrix of sensitivity coefficients has been constructed, principle component analysis (PCA) is an established method for ranking the importance of individual reactions and determining which can be eliminated from the model (Turanyi et al. 1989). An example of the direct application of normalised, local sensitivity analysis and PCA to a nonlinear example model can be found in Additional file 1—Supplementary information Section 2.5.

Degenring et al. (2004) applied this method to a model of the glycolysis and pentose phosphate pathway in *E. coli* (122 parameters and 22 reactions). Employing sensitivity analysis and PCA 49 of the parameters could be discarded from the model whilst retaining an acceptable error bound. Liu et al. (2005) applied an approach using sensitivity analysis, PCA and flux analysis to determine which reactions can be eliminated from a signalling model of the EGRF pathway. They demonstrated that (in one module of the pathway) the number of reactions could be reduced from 85 to 64 whilst retaining a 5% error bound. Smets et al. (2002) used the same approach for a model of gene expression in the *Azospirillum brasilense* Sp7 bacterium. Here, 14 parameters in the full model were reduced to 6 without a substantial loss of accuracy. Apri et al. (2012) introduced an algorithmic derivative-based sensitivity analysis approach to rank parameter importance. The algorithm then attempts to eliminate each parameter in order of sensitivity and gauges the sensitivity of the model output to each elimination. Unfortunately, the resulting reduction was not reliable and demonstrated that local sensitivity analysis is not always sufficient to capture the desired behaviour of the system.

*Global Sensitivity Analysis* Local sensitivity analysis approaches are strongly dependent upon the nonlinearity in the system and the point $\varvec{p}^*$ at which the coefficients are evaluated. The obtained sensitivity coefficient estimates will not necessarily remain accurate far from this point and can give misleading results where nonlinear effects are involved. More statistical approaches that involve sampling large volumes of the parameter space and evaluate the interaction between multiple parameters can lead to more objective estimates of sensitivity. These approaches, known as global sensitivity analysis methods, attempt to establish better estimates of how perturbations in a model’s parameterisation propagate through the system and how they affect the model output.

Estimating global sensitivity indices can be a challenging task, as it is typically not possible to analytically evaluate them. Hence, researchers resort to numerical approaches where, for large systems, such a process can be extremely computationally expensive due to the need to test sensitivity over a large range of parameter space. A wide range of methods to achieve this exist in the literature, as have been reviewed by Zhang and Goutsias (2010), with Monte Carlo sampling being perhaps the most common. Additionally, whilst it does not cover sensitivity analysis’s application to model reduction, Zi (2011) provides a review of sensitivity analysis methods seen in the literature, including a survey of global sensitivity analyses that have been applied to systems biology models and their estimated computational cost.

The use of global sensitivity analysis methods in the reduction of biochemical systems models has seen limited application. Most notably Maurya et al. (2005) introduced a method of multiparametric variability analysis (MPVA) which tests the sensitivity of the objective function in response to multiple parameter changes simultaneously, as opposed to testing a single parameter’s sensitivity at a time. A genetic algorithm (GA)-based approach is then used to search parameter space and find reduced parameter sets that accurately replicate the original dynamics of the output. This approach is demonstrated by application to a 17-dimensional model of the GTPase-cycle module with 48 associated rate parameters. The results show that good agreement can be obtained whilst retaining only 17 parameters. Jayachandran et al. (2014) applied Sobol’s global sensitivity analysis method to three mechanistic models associated with the use of chemotherapy in the treatment of acute lymphoblastic leukaemia. They were able to reduce the number of parameter across the models from 23 to 12. This enabled parameter fitting of these models for individual patients and hence the development of individualised treatment schemes.

#### Optimisation Approaches

An ‘optimisation approach’ here refers to those methods of model reduction that seek to reduce a system by testing a range of ‘candidate’ $\hat{n}$-dimensional reduced models by calculating an associated error metric $\epsilon$ (potentially based upon either a posteriori or a priori information) for each and then selecting the best possible reduction. Of key interest is how the set of candidate reduced models are selected or sampled and what measure of model reduction error is employed in their evaluation. Such methods share a similarity with sensitivity analysis in that they are essentially testing the sensitivity of the error to changes (albeit typically in terms of species as opposed to reactions) in the reduced system.

A large range of optimisation-based reduction approaches have been applied in the context of modelling biochemical reaction networks. Danø et al. (2006) have developed and applied an approach they term elimination of nonessential variables (ENVA). Here the system is simulated where one-by-one each state-variable is eliminated by being fixed at its steady-state value. For a given dimensionality, the reduced model that most accurately reflects the original model dynamics is then returned. The method was applied to a 20-dimensional model of yeast glycolysis where it was able to yield an accurate 6-dimensional reduced model.

Maurya et al. (2005, 2009) develop a method that simultaneously uses a model reduction and a parameter re-estimation algorithm. Here the least influential reaction rates are set to zero to obtain a reduction in the number of reactions. The optimal arrangement for eliminating reactions is expressed as a mixed integer nonlinear programming problem that is solved via a GA. This approach is demonstrated via application to a model of the GTPase-cycle, and it is shown that the original 48 reactions in the system can accurately be reduced to 17 whilst retaining sufficient predictive accuracy. Hangos et al. (2013) highlighted a similar method for the optimal elimination of reactions expressed as a mixed integer quadratic programming problem. Their approach was demonstrated via application to a model of the *Arabidopsis thaliana* circadian clock involving 7 state-variables and 27 reactions. The model was reduced under three cases relating to no light, a constant light source and a pulsing light source. Across these cases they were able to reduce the model by between 1 and 4 parameters whilst retaining an average error in the species dynamics of <6%.

Taylor and Petzold (2008) describe an optimisation approach based upon the ‘parametric impulse phase response curve’ (pIPRC) which essentially describes how the phase of the limit-cycle in an oscillatory model varies in response to changes in parameter values and the error associated with approximating such a cycle. Their reduction methodology is then based upon a minimisation of both the number of state-variables and the pIPRC-associated error such that the reduced model seeks to preserve the oscillation phase. Given these nonlinear constraints the optimisation problem is then solved via a GA that seeks to fix the values of unnecessary state-variables. This approach was demonstrated via application to a 61-dimensional model of the mammalian circadian clock, which was accurately reduced to 13 dimensions whilst incurring only a 5% error in the pIPRC.

Anderson et al. (2011) and Prescott and Papachristodoulou (2012) have developed methods for obtaining an a priori upper bound on the worst-case reduction error under the $L_2$ norm associated with a particular reduced model. In their initial work the estimate required a time-varying linearisation of the system such that an error estimate could be calculated via solving a Lyapunov equation. More recently, a worst-case error bound for the nonlinear system has been developed using the sum of squares decomposition for polynomials. These bounds have been used to develop an optimisation-based method of model reduction. Such an approach will often be faster than other methods as no simulation of the system is required to obtain a metric of reduction accuracy.

### Lumping

Lumping originated as a methodology for the reduction of dynamical systems in the 1960s with the work of Wei and Kuo (1969) and Kuo and Wei (1969). A lumping removes at least one set of state-variables from the system and replaces them with a new dynamical ‘lumped’ variable that represents some direct mapping from the originals. The literature on lumping can be divided into two main categories: (I) those papers that discuss the different types of mapping and their specific properties, and (II) papers that provide algorithms to find a suitable mapping to reduce a given model.

**Fig. 4:**
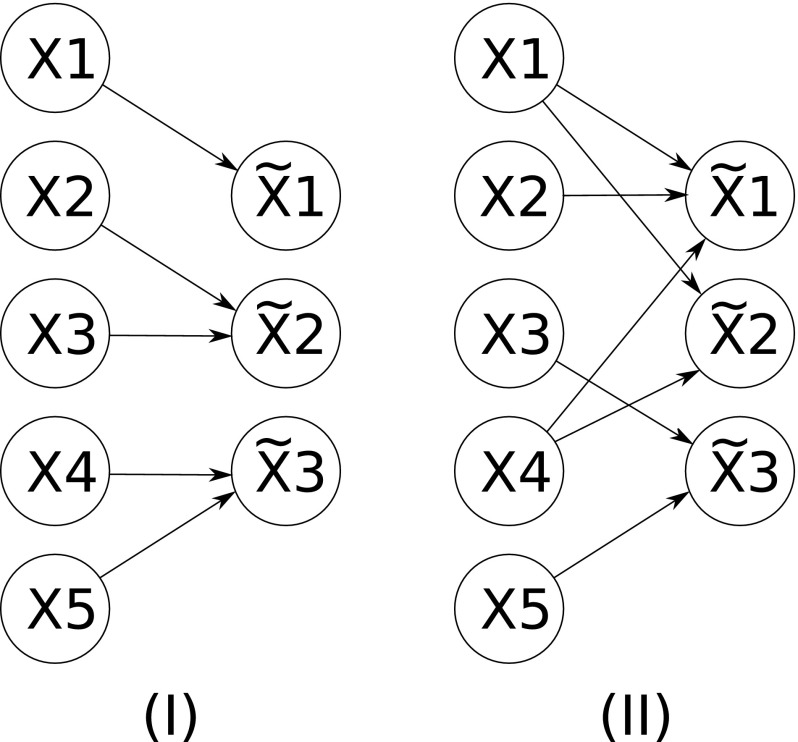
Schematic depiction of proper versus improper lumping. **I** Proper lumping: each of the original species (the *left column*) corresponds to, at most, one of the lumped states (the *right column*). **II** Improper lumping: each of the original states can correspond to one or more of the lumped states

The term lumping is a broadly applicable term that can refer to wide a range of methods; hence, the first set of literature describes the differentiating factors used to specify particular lumping methodologies. These sub-classifications tend to provide constraints on how state-variables may be combined during a reduction and are detailed as follows:

*Proper Versus Improper Lumping* Proper lumping (Wei and Kuo 1969) refers to any scheme where each of the original species appears in only one lumped variable of the reduced model, whilst under improper lumping each of the original species can map to multiple lumped variables (see Fig. 4 for a schematic depiction). An alternative way of understanding proper lumping is as a partitioning of the original species under which each partition can be reduced to a single independent dynamical variable in the kinetics of the reduced model. The majority of lumping methodologies discussed in the literature are proper, which can be constrained so as to maintain some degree of biological interpretability in the reduced network structure.

*Linear Versus Nonlinear Lumping Schemes* Linear lumping (Wei and Kuo 1969; Kuo and Wei 1969) produces lumps that are strictly linear combinations of the original species. Meanwhile, nonlinear schemes (Li et al. 1994a, b; Tomlin et al. 1994) include any lumping that creates lumps via some nonlinear mapping of the original species. The majority of lumping methods discussed in the literature are linear as, similar to proper lumping, such an approach produces reduced networks that are more easily interpreted biologically.

*Exact Versus Approximate Schemes* An exact lumping is one where the dynamics of the reduced system can be exactly mapped to the original dynamics using only new, time-invariant rate parameters (Wei and Kuo 1969; Li et al. 1994a). The conditions for exactness only hold true for a certain subset of lumping schemes and for models with specific properties. As a result, the majority of naive lumping schemes, and most of the lumping methodologies discussed in the literature, will only provide approximate reductions. The issue of how to choose a lumping that will minimise the approximation error comprises the main topic of papers in the literature.

Given the above definitions, the term lumping is generally used to refer to linear, proper lumping in the literature. When applied to systems in the form of Eq. (2), this implies reduction via some linear projection $\varvec{L}\in \left\{ 0,1\right\} ^{\hat{n}\times n}$, where each row of $\varvec{L}$ is pairwise orthogonal. The reduced state-variables $\tilde{\varvec{x}}(t)$ can then be computed as

18 $$\begin{aligned} \tilde{\varvec{x}}(t) = \varvec{L}\varvec{x}(t). \end{aligned}$$

The dynamics of the system now acting upon the reduced variables $\tilde{\varvec{x}}(t)$ can be obtained via application of the Petrov–Galerkin projection as previously outlined. This yields a reduced system of the form

19a $$\begin{aligned} \dot{\tilde{\varvec{x}}}(t)&= \varvec{L}f(\bar{\varvec{L}}\tilde{\varvec{x}}(t),\varvec{p},\varvec{u}(t)) ,\,\,\, \tilde{\varvec{x}}(0)=L\varvec{x}(0)=\tilde{\varvec{x}}_0, \end{aligned}$$

19b $$\begin{aligned} \tilde{\varvec{y}}(t)&= g(\bar{\varvec{L}}\tilde{\varvec{x}}(t),\varvec{p}). \end{aligned}$$

Note that $\bar{\varvec{L}}$ can be any generalised inverse of $\varvec{L}$, and therefore, an infinite number of ways of constructing such a matrix exist. In the original Wei and Kuo papers (Wei and Kuo 1969; Kuo and Wei 1969) outlining linear, proper lumping they suggest selecting the $\bar{\varvec{L}}$ that reconstructs the steady state of the system such that $\varvec{x}^*= \bar{\varvec{L}}\tilde{\varvec{x}}^*$ with $\varvec{x}^*= \lim _{t \rightarrow +\infty } \varvec{x}(t)$. In contrast, Dokoumetzidis and Aarons (2009), following the work of Li and Rabitz (1990), suggest using the Moore–Penrose inverse $\varvec{L}^+$ presumably for the purposes of simplicity and ease of calculation. This choice of lumping inverse, however, can have a significant influence on the model reduction error obtained. An example of the application of linear, proper lumping to a nonlinear example model is given in Additional file 1—Supplementary information Section 2.6.

In recent years, lumping has been used to reduce a number of biochemical systems in the literature. Danø et al. (2006) applied an approach of lumping and subsequent optimisation (which they term LASCO) to a 20-dimensional mode of yeast glycolysis. It was demonstrated that this system could be reduced to 8 dimensions whilst retaining good accuracy. It was also shown that subsequent application of their ENVA reduction approach (as previously outlined) could accurately produce further reductions in the model down to a system of only 3 dimensions that maintained the existence of a Hopf bifurcation.

Dokoumetzidis and Aarons (2009) introduced an algorithmic approach for linear, proper lumping. This is an optimisation-based reduction approach using lumping to obtain candidate reduced models. Their approach seeks to sum two state-variables at each step, testing every possible pair by simulating the resulting reduced model and comparing its output with the original. At each step the pair resulting in the most accurate reduction is lumped, and then the process is repeated a pair at a time. This is continued until the desired reduced dimensionality is reached. Clearly, for large models this can lead to an enormous number of lumpable pairs need to be tested; however, a range of enhancements to reduce the computational burden of this approach were also provided. Much like Danø et al., subsequent parameter optimisation was also suggested to improve the fit of the reduced model to simulated data from the original. This approach was applied to a 26-dimensional model of the NF-$\kappa$B signalling pathway. Reasonable agreement with the original model was retained down to around 13 reduced state-variables, below which the oscillatory behaviour of the system was lost. Gulati et al. (2014) applied the Dokoumetzidis and Aarons methodology to a 62-dimensional model studying the effect of snake venom administration. It was shown that a 5-dimensional model can be produced which reflects the original system dynamics to within a maximal relative error of 20%.

Koschorreck et al. (2007) applied a lumping style approach they termed ‘layer-based reduced modelling’. Finding a lumping under this approach requires a relatively good a priori understanding of the model in order to decompose it into lumpable modules. All components that are strongly connected by a specified class of reactions are considered a ‘layer’ and are subsequently lumped together. Most notably, they apply their approach to a model of an extended subsystem of the insulin signalling pathway, reducing the 24-dimensional system to 11 dimensions with a reduction error ‘within the range of measurement errors in typical experiments’.

Sunnåker et al. (2010, 2011) introduced proper lumping approaches with an emphasis on the ‘zoomability’ of the model, i.e. the ability to switch between particular dimensionalities of reduced models depending upon the application and accuracy desired. This was achieved via use of specific, fractional lumping inverses. In both papers the methods used for finding a suitable lumping have their basis in timescale analysis of the system. In their first paper (Sunnåker et al. 2010) a method was developed to analyse linear systems, under which the system is decomposed into fast and slow species. The algorithm then uses a graph-theoretic approach to analyse the fast part of the system looking for strongly connected components. If found, lumping of the associated species is attempted along with lumping of any linked sink state-variables. This approach is demonstrated via application to a 26-dimensional model of fluorescence emission in photosynthesis, which is reduced to 6 dimensions yielding only a negligible difference in the output profile of the reduced model. In the second paper (Sunnåker et al. 2011) Sunnåker et al. extend their approach to nonlinear models. To find a suitable lumping for a nonlinear system they begin by decomposing the model into fast and slow reactions. Conservation analysis is then applied to the stoichiometry matrix associated only with the fast reactions in the system to find what they term the ‘apparent conservation relations’. Subsets of the variables in these apparent conservation relations are then lumped to produce a reduced model. This methodology is used to reduce a model of glycolysis in *S. cerevisiae* from 9 down to 5 state-variables which still provides an ‘excellent description of the state dynamics’.

### Singular Value Decomposition-Based Model Reduction

Singular value decomposition (SVD) methods are based upon the matrix decomposition of the same name and the resulting lower-rank approximations of matrices it yields. Essentially, the relative magnitude of quantities known as the ‘singular values’ of a matrix determines the extent to which it can be approximated by a matrix of lower rank, and it is this property that is exploited by such methods of model reduction.

SVD implies that any $m\times n$ matrix $\varvec{A}$ can be decomposed into the form

20 $$\begin{aligned} \varvec{A} = \varvec{U}\varvec{{\varSigma }} \varvec{V}^*, \end{aligned}$$

with $\varvec{U}$ an $m\times m$ matrix, $\varvec{{\varSigma }}$ an $m \times n$ diagonal matrix , and $\varvec{V}^*$ an $n\times n$ matrix. Under such a decomposition, the *m* diagonal entries $\sigma _i$ of $\varvec{{\varSigma }}$ are referred to as the singular values of $\varvec{A}$.

Via the Eckart–Young–Mirsky theorem Eckart and Young (1936), the SVD provides a way to approximate $\varvec{A}$ with a lower-rank matrix $\tilde{\varvec{A}}$. If a reduced approximation of rank $\hat{n}$ is sought, such that $\text {Rank}\left( \tilde{\varvec{A}}\right) = \hat{n}$, this can be computed as

21 $$\begin{aligned} \tilde{\varvec{A}} = \varvec{U}_1\tilde{\varvec{{\varSigma }}}\varvec{V}_1^*, \end{aligned}$$

where that $\tilde{\varvec{{\varSigma }}} = \text {diag}\left( \sigma _1,\ldots ,\sigma _{\hat{n}} \right)$, and $\varvec{U}$ and $\varvec{V}^*$ have been partitioned such that

22 $$\begin{aligned} \varvec{U} = \left( \begin{array}{cc} \varvec{U}_1&\varvec{U}_2 \end{array}\right) , \, \varvec{V}^*= \left( \begin{array}{c} \varvec{V}^*_1 \\ \varvec{V}^*_2 \end{array}\right) . \end{aligned}$$

It is this approximation of a matrix by one of lower rank that is exploited by SVD-based methods of model reduction.

#### Balanced Truncation

**Fig. 5:**
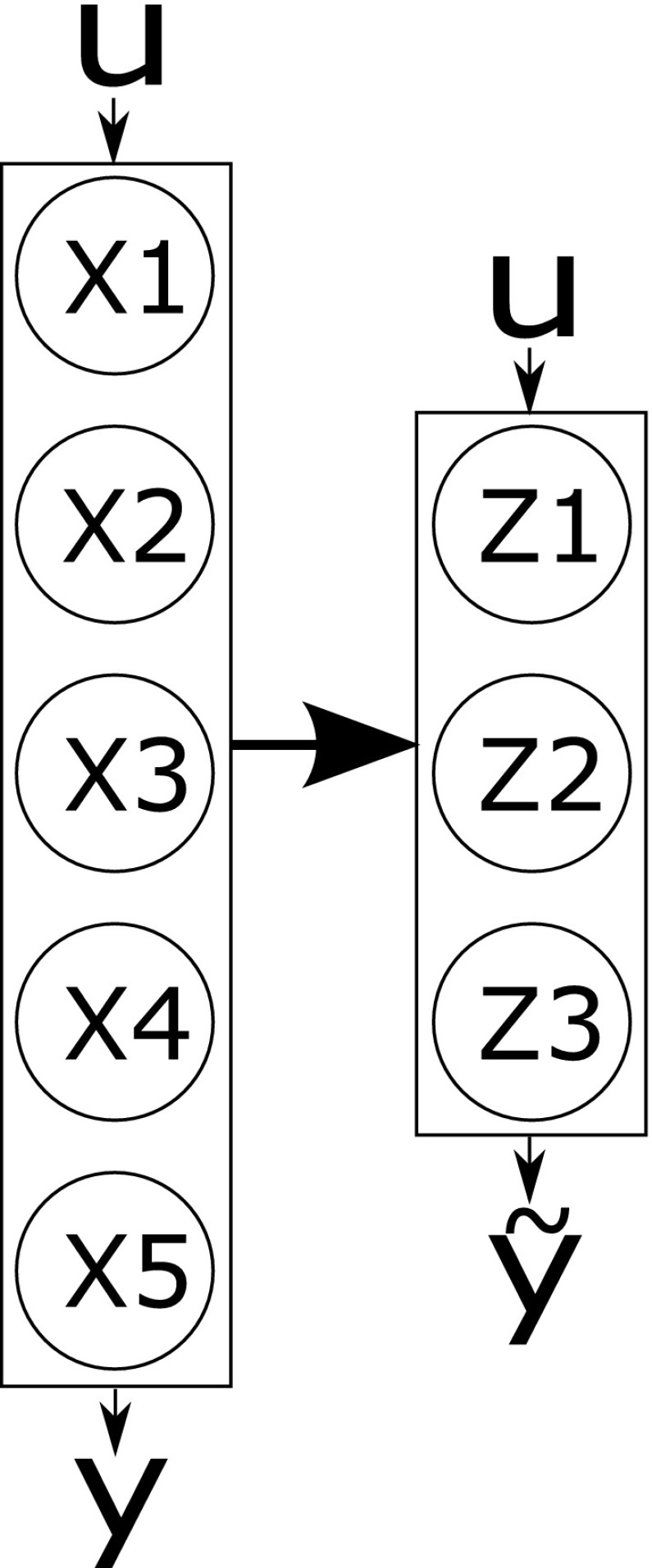
Model reduction via balanced truncation. The method seeks to reduce a system whilst preserving the input–output relationship of the model. This is achieved via a coordinate transformation of the state-variables

One SVD method that has been employed in the reduction of biochemical systems is that of balanced truncation (Liebermeister 2005; Liebermeister et al. 2005; Meyer-Bäse and Theis 2008). The method is most commonly used in the field of control theory and was originally devised in the early 1980s (Moore 1981). It was subsequently refined by a number of authors and has become a well-developed methodology covered in many textbooks on control theory (Skogestad and Postlethwaite 2005; Dullerud and Paganini 2000). It is applicable to controlled models in a state-space representation form and focuses on reducing systems whilst preserving the overall input–output behaviour of the model. Typically, the method is used for the simplification of time-invariant, linear systems and does not rely upon timescale separation of fast and slow processes (Fig. 5).

Crucially, balanced truncation seeks to exploit the concepts of controllability (how strongly each of the state-variables responds to changes in the input) and observability (how strongly the output responds to changes in the state-variables). To quantify these concepts it is possible to construct a pair of matrices known as the controllability and observability Gramians. Balanced truncation seeks a ‘balancing’ transformation of the state-variables under which these Gramians are equalised and diagonalised. This implies that the transformed state-variables are orthogonal in the input–output space of the model and those contributing least to the overall input–output relationship can therefore be truncated without impacting the remaining variables.

In the linear case, balanced truncation begins with a controlled system of the form

$$\begin{aligned} \dot{\varvec{x}}&= \varvec{A}\varvec{x} + \varvec{B}\varvec{u}, \\ \varvec{y}&= \varvec{C}\varvec{x}. \end{aligned}$$

The controllability and observability Gramians, $\varvec{\mathcal {P}}$ and $\varvec{\mathcal {Q}}$, respectively, can then be obtained by solving the Lyapunov equations

$$\begin{aligned} \varvec{A}\varvec{\mathcal {P}} + \varvec{\mathcal {P}}\varvec{A}^\intercal +\varvec{B}\varvec{B}^\intercal =0, \,\,\text {and}\,\, \varvec{A}^\intercal \varvec{\mathcal {Q}} + \varvec{\mathcal {Q}}\varvec{A}+\varvec{C}^{\intercal }\varvec{C}=0. \end{aligned}$$

The aim is then to find a balancing transformation which, when applied to the state-variables, equalises and diagonalises both $\varvec{\mathcal {P}}$ and $\varvec{\mathcal {Q}}$. Such a transformation can be obtained via the following steps; first, perform a Cholesky factorisation of both of the Gramians to give

$$\begin{aligned} \varvec{\mathcal {P}} = \varvec{L}^\intercal \varvec{L}, \,\,\text {and} \,\, \varvec{\mathcal {Q}} = \varvec{R}^\intercal \varvec{R}. \end{aligned}$$

Now take a singular value decomposition of the newly formed matrix $\varvec{L}\varvec{R}^\intercal$ to obtain

$$\begin{aligned} \varvec{L}\varvec{R}^\intercal =\varvec{U} \varvec{{\varSigma }} \varvec{V}^\intercal , \end{aligned}$$

using this, the balancing transformation $\varvec{T}$ and its inverse $\bar{\varvec{T}}$ can be computed as

$$\begin{aligned} \varvec{T} = \varvec{{\varSigma }}^{-\frac{1}{2}}\varvec{V}^\intercal \varvec{R} \,\,\text {and} \,\, \bar{\varvec{T}} = \varvec{L}^\intercal \varvec{U}\varvec{{\varSigma }}^{-\frac{1}{2}}. \end{aligned}$$

Given a reduced dimensionality $\hat{n}$ the reduced model can be constructed via the following transformations

$$\begin{aligned} \varvec{x} \rightarrow \tilde{\varvec{x}}&= \varvec{P}\varvec{T}\varvec{x}, \\ \varvec{A} \rightarrow \tilde{\varvec{A}}&= \varvec{P}\varvec{T}\varvec{A}\bar{\varvec{T}}\varvec{P}^{\intercal },\\ \varvec{B} \rightarrow \tilde{\varvec{B}}&= \varvec{P}\varvec{T}\varvec{B},\\ \varvec{C} \rightarrow \tilde{\varvec{C}}&= \varvec{C}\bar{\varvec{T}}\varvec{P}^{\intercal }, \end{aligned}$$

where $\varvec{P}$ is an $\hat{n}\times n$ matrix of the form $\varvec{P} = \left[ \varvec{I}_{\hat{n}} \, \varvec{0}\right]$. This gives a reduced, $\hat{n}$-dimensional model of the form

$$\begin{aligned} \dot{\tilde{\varvec{x}}}&= \tilde{\varvec{A}}{\tilde{\varvec{x}}} + \tilde{\varvec{B}}\varvec{u}, \\ \tilde{\varvec{y}}&= \tilde{\varvec{C}}\tilde{\varvec{x}}. \end{aligned}$$

Such an approach has a number of strengths, especially in the construction of highly reduced systems that will provide an accurate approximation of output for any given input values. Additionally the method provides the ability to construct an a priori error bound for a given reduction based upon the singular values of the balanced Gramian (known as the Hankel singular values). Unfortunately, the transformation applied to the state-variables will typically mask the biological interpretability of the reduced dynamical system, and as such, balanced truncation can be considered as a black-box approach to model reduction.

Balanced truncation was originally devised for the reduction of linear systems; however, in recent years generalisations for nonlinear cases have emerged (Härdin and van Schuppen 2006; Lall et al. 2002; Hahn and Edgar 2000, 2002). For nonlinear systems, however, the Gramians computed are typically only an approximation. Given the usually nonlinear nature of biochemical models it is these methods that may possess the most relevance. In particular, empirical balanced truncation, which constructs approximate Gramians via repeated numerical simulations of the model under perturbations, may be highly applicable within the context of biochemical systems but has not yet seen published use. An example of the application of linearisation and balanced truncation to a nonlinear example model is given in Additional file 1—Supplementary information Section 2.7.

In the biochemical modelling literature balanced truncation has seen relatively limited application. Liebermeister et al. (2005) outlined an approach that involved partitioning a model into two sets of species: a ‘core’ set containing the species and reactions of primary interest to the modeller and an ‘environmental’ set of terms present in the model, but of little interest. The approach then seeks to linearise and apply balanced truncation to the set of environmental species in order to construct a reduced model. This method was applied to a model of glycolysis from the KEGG database. A particular 3- dimensional sub-module was chosen to represent the core set, and the remaining 20 interacting species were found to be environmental relative to these dynamics of interest. It was demonstrated that this environmental set could be reduced to a single state-variable whilst retaining an accurate description of the core dynamics.

Härdin and van Schuppen (2006) demonstrate a similar approach of system linearisation followed by balanced truncation to a model of yeast glycolysis. They showed that a 13-dimensional model could be reduced to 3 state-variables. Unfortunately, whilst the application of balanced truncation incurred very little error, the initial linearisation step was shown to suffer a prohibitive error cost.

Sootla and Anderson (2014) developed a method of balanced truncation for application to linearised systems. To avoid issues of biological interpretability, they impose the condition that Gramians must be block diagonal, hence preserving meaning between sub-modules, with the interior of modules reduced by a balancing transformation. Their method requires that the system is monotone in order to obtain such block diagonal Gramians.

### Miscellaneous Methods

There are a range of model reduction methods described in the literature that do not sit comfortably within any of the areas so far covered in this review. The following section provides a brief overview of these methods.

*Motif Replacement* Such approaches decompose a system into various interconnected sub-modules that can be replaced by simpler motifs. Typically this requires a relatively high degree of heuristic insight in order to spot replacement motifs. Conzelmann et al. (2004) developed a motif replacement method where the model is initially decomposed into a number of sub-modules, and each module is then treated in isolation. Reactions feeding into a sub-module are considered as inputs, and those exiting in the sub-module are considered outputs. Each sub-module is then simulated under perturbations of its inputs in order to construct an overall input–output profile. Comparison of the input–output profiles with each other and standard profile types from signal theory can be used to replace the modules with simpler motifs that replicate their behaviour. The method was demonstrated via application to a model of EGF receptor signalling enabling the accurate reduction of several sub-modules. A similar approach of partitioning a biochemical network into sub-modules and applying motif replacement based upon their input–output profiles was also briefly discussed by Vallabhajosyula and Sauro (2006).

*Reduction Workflow* This topic concerns the general heuristics used to guide the application of model reduction methods.

Quaiser et al. (2011) propose an approach whereby a model is reduced iteratively until the system is sufficiently identifiable, i.e. until the variances associated with the parameter estimates are sufficiently small. This method was demonstrated via application to a model of JAK–STAT signal transduction. Over 6 reduction steps the number of state-variables was reduced from 17 to 10 and the number of parameters from 25 to 10, at which point the model parameters could be accurately estimated given a limited set of input–output data.

Apri et al. (2014) propose an iterative heuristic for obtaining a reduced model. Given a system in the form of (2), with experimental results that can be treated as outputs and experimental conditions that can be treated as inputs, the approach is twofold. Firstly, model reduction is performed via an iterative algorithm involving state-variable and parameter truncation, lumping, and the re-fitting of parameters. Reduction is repeatedly applied until the reduced model cannot capture the experimental behaviour within an adequate error bound. Secondly, model ‘discrimination’ is performed to determine the experimental conditions (within a feasible range) that maximise the error between the reduced and original models. If the maximal error exceeds the previously defined limit, then new experimental data obtained under the error-maximising conditions are included and the reduction step is rerun. These steps are applied recursively until a reduced model is obtained that adequately captures the results under all possible experimental conditions. The method is demonstrated via application to two systems: firstly a model of a genetic interaction network in flower development of *A thaliana* where it is shown that a reduction from 37 to 31 parameters still maintains accuracy for all reasonable experimental conditions, and secondly, to a model of the EGFR signalling pathway where it is shown that a reduction from 23 to 17 state-variables and 50–25 kinetic parameters was sufficient to yield no more than a 25% error for all possible experimental conditions.

Maiwald et al. (2016) present a heuristic for reduction whereby a model is reduced until it is identifiable relative to the experimental data available. This is achieved by evaluating parameter profile likelihoods and then seeking to reduce reactions associated with the least identifiable parameters. Structurally non-identifiable parameters can, at least theoretically if not practically, be eliminated from the system via the exploitation of intrinsic symmetries in the system. In the case of the weakly identifiable parameters in the system, associated reactions are reduced via approaches such as lumping, deletion of species, and algebraic replacement until an identifiable system is obtained.

*Reducing Combinatorial Complexity* Particular attention can be given to model reduction in the context of combinatorially complex systems such as those found in the modelling of scaffold proteins. Such proteins have a large number of binding sites and can form complexes in many different combinations. Using a standard modelling approach each possible binding configuration is considered a separate species and its concentration is modelled as such. Clearly this can lead to a combinatorial explosion in the number of state-variables, and hence, there exist a number of methods of model reduction which seek to alleviate this complexity. Borisov et al. (2005) demonstrated a model reduction approach for such systems via a transformation of the possible states into ‘macro-states’, effectively improper lumpings of the original terms. However, this work only applies to scaffold proteins with independent binding sites or with only one controlling domain. Subsequently, Conzelmann et al. (2006, 2008) extended this approach to more general models of scaffold protein interactions (or models with similar combinatorially complex interactions). A hierarchical state-variable transformation is introduced; this transformation is guided a form of sensitivity analysis under the assumption that many of the possible complexes will have a limited effect on the outputs of interest.

*Further Approaches* Rao et al. (2013, 2014) developed an approach that seeks to reduce the set of chemical equations defining a biochemical reaction network via an iterative process of equilibrating and deleting one complex (as defined under chemical reaction network theory Feinberg 1987) at a time. This approach is applied using an optimisation algorithm until a pre-defined error tolerance is reached. The method is demonstrated via application to a model of yeast glycolysis where it was found that deletion of 4 complexes (producing a reduction from 12 state-variables, 88 parameters and 12 reactions to 7 state-variables, 50 parameters and 7 reactions) incurred a <8% average error across time and state-variables. A model of fatty acid beta oxidation was also considered where the deletion of 14 complexes (corresponding to a reduction from 42 state-variables to 29) could be obtained incurring an average error of 7.5%.

Whiteley (2010) applies an approach of mesh refinement via a posteriori error analysis, commonly used in improving the numerical simulation of partial differential equations via finite element methods, to the reduction of biochemical systems. Via an iterative process, this approach determines which state-variables should be retained and which can be fixed (beginning with the ‘all fixed’ possibility) within each time-interval to meet some pre-assigned error bound.

Transtrum and Qiu (2016) outline an approach based on differential geometry known as the manifold boundary approximation method. This approach allows the construction of a model manifold $\mathcal {M}$ describing the parameter-dependent variation in certain pre-defined outputs or ‘quantities of interest (QoIs)’. By repeatedly evaluating the Fisher information matrix it is typically possible to construct geodesics along $\mathcal {M}$ that can be used to define boundaries in parameter space. These boundaries imply that at certain positions in parameter space the QoIs can be captured by a reduced system. Using this information it is possible to construct reduced systems in these spaces by allowing certain combinations of parameters to tend to infinity or zero. In the paper it is demonstrated that this approach can recover the QSSA for the Michaelis–Menten enzyme–substrate reaction model. They also demonstrate the methods application to a 15-dimensional model of ERK activation via the interacting EGF and NGF pathways. Here they recover models in various states of reduction depending upon the specific QoIs—notably, they demonstrate that a 6-dimensional network can describe the overall input output behaviour of EGF, NGF and their effect on ERK.

Finally, Schmidt et al. (2008) develop a method for reducing complexity in individual rate expressions that can be expressed as a rational function, i.e. the ratio of two polynomials. The method employs the notion of identifiability—recall that if an expression is unidentifiable, it implies that another parameter set can be used to produce the same dynamic behaviour. Exact reduction can often be obtained via exploiting linear dependencies arising from unidentifiability of reaction rates for simulated data sets. This can be exploited further to obtain an approximate reduction by discarding those terms in the rate expression that contribute least to the reaction.

## Discussion

There exists no one-size-fits-all method of model reduction which can be considered optimal for all large-scale biochemical systems irrespective of the context in which it is applied. Indeed, the ‘best’ reduced model that can be obtained for a particular system is inextricably linked to both the overall aims of the modeller, the scope and scale of the of the approximation error they are willing to incur, and the nature of the model they are seeking to reduce.

This review defined a method of reduction as any approach seeking to approximate the dynamics of a given model by a simpler system, featuring a smaller number of reactions or reactants. As was shown, even given this relatively narrow definition, methods for the reduction of biochemical systems can take a wide number of forms. Table 1 provides an overview of the main methods of model reduction reviewed within this paper and their attributes.

**Table 1:** Comparison of methods of model reduction for biochemical reaction networks

	Suitable for very high-dimensional systems	Suitable for stiff systems	Nonlinear systems	Preserves species meanings
Coordinate preserving timescale methods	–	$\checkmark$	$\checkmark$	$\checkmark$
Coordinate transforming timescale methods	–	–	$\checkmark$	$\times$
Sensitivity analysis	–	–	–	$\checkmark$
Optimisation approaches	$\checkmark$	$\checkmark$	$\checkmark$	$\checkmark$
Lumping	$\checkmark$	$\checkmark$	$\checkmark$	–
Balanced truncation	$\checkmark$	–	–	$\times$

$\checkmark$ Implies a method is suitable for this context, – implies certain variants are suitable for this context and others are not, and $\times$ implies a method is not suitable for this context

Timescale exploitation methods are particularly applicable where reactions in the system occur across a wide range of timescales (typically dictated by widely varying reaction rate constants) or the modeller wishes to access a reduced model that is accurate within a particular time-interval. Coordinate preserving timescale exploitation methods usually require that the species of the system can be explicitly defined as either fast or slow. Where this is possible, it enables access to intuitively understood reductions of the system. Coordinate transforming timescale exploitation methods can be used in a more general setting and will often produce more accurate reductions, but the biological meaning of the reduced model can be somewhat obscured by the change of variables.

Optimisation- and sensitivity analysis-based approaches to model reduction are the most intuitive of the methods reviewed here. These approaches can be applied to any model in general, but can be highly computationally expensive for large models where the parameter space to be searched and simulated is often prohibitive.

Lumping is a broad class of model reduction, but in its common definition of linear, proper lumping it represents a highly algorithmic and relatively intuitive methodology. However, the question of how the best lumping is determined for a nonlinear system is still somewhat open—approaches in the literature often rely upon trial and error, which can be computationally expensive for very large systems.

SVD methods represent some of the more esoteric methods that can be applied. They apply transformations to the state-variables that typically produce transformed variables with an obscured biological meaning. However, these methods work especially well when a model can be treated as a black-box and only the input–output behaviour is of interest to the modeller. These methods can often produce very accurate and low-dimensional reductions.

The relatively recent advent of systems biology has produced a wealth of highly detailed models, providing great insight into the mechanistic underpinnings of physiological systems. It seems inevitable that researchers in both academia and industry will increasingly seek to use these models in new ways beyond exploratory research. As they do so, the perennial issue of complexity will be necessarily brought into focus again. In those areas of science, such as engineering, most used to pragmatic compromise in the face of systemic complexity, methods of model reduction are already a well-utilised tool of research. Hence model reduction techniques, such as those introduced throughout this review, must also become a more familiar tool in the biochemical modeller’s arsenal.

Whilst such methods have the potential to provide substantial benefits, enabling previously intractable problems to be tackled and allowing modellers to extract insight from complexity, their application should never be considered a ‘magic bullet’. Reduced systems typically only remain valid within a specific region of parameter space or predictive for a set of pre-defined outputs. Even in archetypal examples such as the QSSA being applied to the enzyme–substrate equation, validity is only guaranteed for particular model parameterisations and, when used inappropriately, can lead to the loss of dynamical phenomena in the original system (Flach and Schnell 2006). In general, model reduction can therefore be thought of as a trade between the simplicity of the reduced model and the predictive power that it retains. Hence, before applying such methods, it is important to be clear on how the reduced model will be used, the specific questions you are aiming to answer, and how the reduction method should be constrained in terms of loss of information.

The development and application of model reduction methods for the field of systems biology remain an ongoing and active area of research. There are a number of likely ways forward including the combining of existing methodologies, the further tailoring of methods to a biological context, and study of the relationship between model reduction and parameter identifiability. Methods from other fields, such as those based upon proper orthogonal decomposition and Krylov subspaces (Antoulas 2005), might also find specific applications in this setting.

## Electronic supplementary material

Below is the link to the electronic supplementary material.

Supplementary material 1 (pdf 536 KB)

## Abbreviations

CSP: Computational singular perturbation
DQSSA: Delay quasi-steady-state approximation
ENVA: Elimination of nonessential variables
GA: Genetic algorithm
LASCO: Lumping and subsequent optimisation
ILDM: Intrinsic low-dimensional manifold method
MPVA: Multiparametric variability analysis
PCA: Principle component analysis
QSSA: Quasi-steady-state approximation
REA: Rapid equilibrium approximation
SVD: Singular value decomposition
ZDP: Zero-derivative principle
