# Supplementary material for: Methods of Model Reduction for Large-Scale Biological Systems: A Survey of Current Methods and Trends
Source: Bull Math Biol. 2017 Jun 27;79(7):1449–86. doi: 10.1007/s11538-017-0277-2 (PMC5498684; doi:10.1007/s11538-017-0277-2)
Supplement: Supplementary file 1 — Supplementary material 1 (pdf 536 KB) [file 11538_2017_277_MOESM1_ESM.pdf]

Methods of model reduction for large-scale biological systems – supplementary information

Thomas J. Snowden[1,2], Piet H. van der Graaf[2,3], Marcus J. Tindall[1,4,*]
1 Department of Mathematics and Statistics, University of Reading, Reading, UK
2 Certara QSP, University of Kent Innovation Centre, CT2 7FG Canterbury, UK
3 Leiden Academic Centre for Drug Research, Universiteit Leiden, Leiden, Netherlands
4 The Institute for Cardiovascular and Metabolic Research (ICMR), University of Reading, Reading, UK
∗ E-mail: M.Tindall@reading.ac.uk

Contents

1 Supplementary information 1
1.1 The Petrov-Galerkin projection . . . 1
1.2 The zero-derivative principle . . . 2
1.3 The intrinsic low dimensional manifold method . . . 3
1.4 Computational singular perturbation . . . 3
1.5 Local sensitivity analysis . . . 5

2 A nonlinear example system 6
2.1 Conservation analysis . . . 8
2.2 Model Linearisation . . . 11
2.3 Coordinate Preserving Timescale Exploitation . . . 12
2.4 Coordinate Transforming Timescale Exploitation . . . 13
2.5 Sensitivity Analysis . . . 16
2.6 Lumping . . . 18
2.7 Singular value decomposition based methods . . . 18

1 Supplementary information

1.1 The Petrov-Galerkin projection

Methods of model reduction can be considered as a projection of the state-variables to a lower dimensional subspace $\mathcal{V}: \ \dim(\mathcal{V}) = \hat{n}$ of the original phase-space, within which some relevant set of the system's trajectories can be adequately approximated. Mathematically, applying such a projection to obtain a reduced dynamical system is underpinned by the Petrov-Galerkin projection [1] which will be detailed here.

Consider a basis $\boldsymbol{B}$ of the subspace $\mathcal{V}$ such that $\boldsymbol{B} = [\boldsymbol{b}_1, \ldots, \boldsymbol{b}_{\hat{n}}] \in \mathbb{R}^{n\times\hat{n}}$. Assuming $\boldsymbol{B}$ has been selected such that it provides an adequately accurate approximation of the original states $\boldsymbol{x}(t)$ within the subspace $\mathcal{V}$, then

$$\boldsymbol{x}(t) \approx \boldsymbol{B}\tilde{\boldsymbol{x}}(t) \tag{1}$$

with $\tilde{\boldsymbol{x}}(t) \in \mathbb{R}^{\hat{n}}$ representing the reduced set of state-variables. Substituting this approximation into the stoichiometric model form yields

$$\boldsymbol{B}\dot{\tilde{\boldsymbol{x}}}(t) = \boldsymbol{f}(\boldsymbol{B}\tilde{\boldsymbol{x}}(t)) + \boldsymbol{g}(\boldsymbol{B}\tilde{\boldsymbol{x}}(t))\boldsymbol{u}(t) + \boldsymbol{\rho}(t) \tag{2}$$

where it is assumed that $\boldsymbol{B}$ is time-invariant. Additionally, $\boldsymbol{\rho}(t) \in \mathbb{R}^n$ is termed the residual and addresses the discrepancy emerging from the fact that $\boldsymbol{B}\tilde{\boldsymbol{x}}$ is typically not an exact solution of the system for all times.

Now let $\mathcal{W}$ represent a subspace that is orthogonal to the residual $\boldsymbol{\rho}(t)$ with a basis $\boldsymbol{C} \in \mathbb{R}^{n\times\hat{n}}$ such that $\boldsymbol{C}^\intercal\boldsymbol{\rho}(t) = 0$. Hence, left multiplying equation (2) by $\boldsymbol{C}^\intercal$ produces

$$\boldsymbol{C}^\intercal\boldsymbol{B}\dot{\tilde{\boldsymbol{x}}}(t) = \boldsymbol{C}^\intercal\boldsymbol{f}(\boldsymbol{B}\tilde{\boldsymbol{x}}(t)) + \boldsymbol{C}^\intercal\boldsymbol{g}(\boldsymbol{B}\tilde{\boldsymbol{x}}(t))\boldsymbol{u}(t) \tag{3}$$

Assuming $\boldsymbol{C}^\intercal\boldsymbol{B}$ is non-singular, this finally leads to a reduced dynamical system of the form

$$\dot{\tilde{\boldsymbol{x}}}(t) = \left(\boldsymbol{C}^\intercal\boldsymbol{B}\right)^{-1}\boldsymbol{C}^\intercal\boldsymbol{f}(\boldsymbol{B}\tilde{\boldsymbol{x}}(t)) + \left(\boldsymbol{C}^\intercal\boldsymbol{B}\right)^{-1}\boldsymbol{C}^\intercal\boldsymbol{g}(\boldsymbol{B}\tilde{\boldsymbol{x}}(t))\boldsymbol{u}(t). \tag{4}$$

This simplification of a dynamical system to a lower dimensional subspace is known as the Petrov-Galerkin projection. In the special case where $\boldsymbol{B} = \boldsymbol{C}$ it is known simply as the Galerkin projection. In that case

$$\left(\boldsymbol{B}^\intercal\boldsymbol{B}\right)^{-1}\boldsymbol{B}^\intercal = \bar{\boldsymbol{B}} \tag{5}$$

Such that $\bar{\boldsymbol{B}}$ is a generalised left-inverse of $\boldsymbol{B}$ and $\bar{\boldsymbol{B}}\boldsymbol{B} = \boldsymbol{I}_{\hat{n}}$ (the $\hat{n}$ dimensional identity matrix).

Whilst the explanation given above provides an explanation of how to apply a Petrov-Galerkin projection, it does not provide a methodology for finding suitable bases $\boldsymbol{B}$ and $\boldsymbol{C}$ for a given model. It is methodologies of this kind that comprise the majority of the model reduction literature.

1.2 The zero-derivative principle

The zero-derivative principle (ZDP) method of model reduction essentially provides a computational approach for extending the QSSA to a higher order approximation. To understand the derivation of this method observe that a model of a biochemical reaction network can be expressed in the form

$$\dot{\boldsymbol{x}}(t) = \boldsymbol{f}(\boldsymbol{x}(t)), \tag{6}$$

where $\boldsymbol{f}(\boldsymbol{x}(t)) = \sum_{i=1}^{m}\boldsymbol{s}_{ci}v_i(\boldsymbol{x}(t))$ with $\boldsymbol{s}_{ci}$ referring to the ith column of the stoichiometry matrix $\boldsymbol{S}$.

If it is now assumed that the species $\boldsymbol{x}(t)$ can be partitioned into a slow group $\boldsymbol{x}_s(t)$ and a fast group $\boldsymbol{x}_f(t)$, as in the case of the generalised QSSA described above, then the ν-th order ZDP employs the set of algebraic conditions

$$\frac{\mathrm{d}^i\boldsymbol{x}_f}{\mathrm{d}t^i} = 0, \;\; i = 1, \ldots, \nu. \tag{7}$$

Consider, for example, the first order ZDP; here equation (7) yields the algebraic equations

$$0 = \frac{\mathrm{d}\boldsymbol{x}_f}{\mathrm{d}t} = \boldsymbol{f}_f \tag{8a}$$

$$0 = \frac{\mathrm{d}^2\boldsymbol{x}_f}{\mathrm{d}t^2} = \frac{\partial\boldsymbol{f}_f}{\partial\boldsymbol{x}_s}\boldsymbol{f}_s + \frac{\partial\boldsymbol{f}_f}{\partial\boldsymbol{x}_f}\boldsymbol{f}_f \tag{8b}$$

and can be similarly extended for higher orders of the ZDP. Clearly, from equation (8a) the zeroth order ZDP is equivalent to the QSSA.

To achieve a reduction the aim is to solve equations (7) for the fast species $\boldsymbol{x}_f$ and substitute the solutions into the slow dynamics $\dot{\boldsymbol{x}}_s(t) = \boldsymbol{f}_s$, hence decoupling the system. Unfortunately, the first order and higher ZDP approximations cannot often be solved analytically. As a result, reductions of this type often employ numerical solutions of equations (7) to achieve a simplification. As a result, this form of reduction often comes at the cost of obscuring the intuitiveness of the reduced model.

1.3 The intrinsic low dimensional manifold method

The intrinsic low dimensional manifold method (ILDM) method offers an approach for obtain an eigenbasis decomposition of the system, whilst avoiding the potential numerical issues typically associated with tightly clustered or repeated eigenvalues. The method takes a linearised system in and applies a Schur decomposition to the Jacobian J to obtain an upper triangular matrix U, such that

$$\boldsymbol{U} = \boldsymbol{Q}^{-1}\boldsymbol{J}\boldsymbol{Q} = \begin{bmatrix} \boldsymbol{U}_s & \boldsymbol{U}_c \\ 0 & \boldsymbol{U}_f \end{bmatrix}. \tag{9}$$

Here, Givens rotations have been applied to reorder $\boldsymbol{U}$ in terms of the descending eigenvalues of $\boldsymbol{J}$, and hence in terms of the slow and fast timescales. Additionally it has been assumed that the slow ($\boldsymbol{\lambda}_s$) and fast ($\boldsymbol{\lambda}_s$) eigenvalues are divided by a substantial eigengap (such that $0 > \min\{\mathrm{Re}(\boldsymbol{\lambda}_s)\} \gg \max\{\mathrm{Re}(\boldsymbol{\lambda}_f)\}$), and hence that a meaningful timescale decomposition is attainable.

Here the block upper triangular form $\boldsymbol{U}_f$ corresponds to the fast eigenvalues, $\boldsymbol{U}_s$ to the slow eigenvalues, and the $\boldsymbol{U}_c$ block couples the fast and slow dynamics of the system under a Schur basis [2]. The method then aims to decouple the matrix (i.e. eliminate the $\boldsymbol{U}_c$ terms) by finding the solution $\boldsymbol{Y}$ of the Sylvester equation

$$\boldsymbol{U}_{\text{slow}}\boldsymbol{Y} - \boldsymbol{Y}\boldsymbol{U}_{\text{fast}} = \boldsymbol{U}_{\text{coup}}. \tag{10}$$

By defining

$$\boldsymbol{T} = \boldsymbol{Q}\left(\boldsymbol{I} + \begin{bmatrix} 0 & \boldsymbol{Y} \\ 0 & 0 \end{bmatrix}\right) \text{ and } \boldsymbol{T}^{-1} = \left(I - \begin{bmatrix} 0 & \boldsymbol{Y} \\ 0 & 0 \end{bmatrix}\right)\boldsymbol{Q}^{-1}, \tag{11}$$

it is therefore possible to obtain

$$\boldsymbol{T}^{-1}\boldsymbol{J}_{\boldsymbol{x}_c}\boldsymbol{T} = \begin{bmatrix} \boldsymbol{U}_{\text{slow}} & 0 \\ 0 & \boldsymbol{U}_{\text{fast}} \end{bmatrix}. \tag{12}$$

The transformation $\boldsymbol{T}$ can be then be applied to the original set of state-variables $\boldsymbol{x}(t)$ such that

$$\boldsymbol{x} \to \bar{\boldsymbol{x}} : \bar{\boldsymbol{x}} = \boldsymbol{T}^{-1}\boldsymbol{x} = \begin{pmatrix} \bar{\boldsymbol{x}}_{\text{slow}} \\ \bar{\boldsymbol{x}}_{\text{fast}} \end{pmatrix}, \tag{13}$$

and hence, via the Petrov-Galerkin projection as previously introduced, this transformation of the linearised system can be applied for the reduction of the original nonlinear model, yielding

$$\dot{\bar{\boldsymbol{x}}}(t) = \boldsymbol{T}^{-1}\boldsymbol{S}\boldsymbol{v}\left(\boldsymbol{T}\bar{\boldsymbol{x}}(t), \boldsymbol{k}\right). \tag{14}$$

As in other examples of timescale exploitation, it is now possible to truncate the fast state-variables from this transformed system leaving only a reduced, approximate model for the slow dynamics of the system.

1.4 Computational singular perturbation

To understand the application of the CSP method, begin with a system of the form

$$\dot{\boldsymbol{x}}(t) = \boldsymbol{f}(\boldsymbol{x}(t)) \tag{15}$$

which is related to the stoichiometric model formulation via

$$\boldsymbol{f}(\boldsymbol{x}(t)) = \sum_{i=1}^{m} \boldsymbol{s}_{ci} v_i(\boldsymbol{x}(t)), \tag{16}$$

with $\boldsymbol{s}_{ci}$ referring to the ith column of the stoichiometry matrix $\boldsymbol{S}$. The goal of CSP is then to search for a change of basis for the vector field $\boldsymbol{f}$.

For example, let $\boldsymbol{A}(t)$ be an $n \times n$ matrix whose columns ($\boldsymbol{a}_i(t)$ for $i = 1, \ldots, n$) form a basis for $\mathbb{R}^n$ at any given time point t, such that $\boldsymbol{f}$ can be represented as

$$\boldsymbol{f} = \boldsymbol{A}(t)\boldsymbol{g} \tag{17}$$

with $\boldsymbol{g}$ the new coordinates for the transformed vector field. Define $\boldsymbol{B}(t) = \boldsymbol{A}(t)^{-1}$ and let $\boldsymbol{b}_j(t)$ for $j = 1, \ldots, n$ represent the rows of B, such that

$$\boldsymbol{g} = \boldsymbol{B}(t)\boldsymbol{f}. \tag{18}$$

Upon differentiating equation (18) with respect to time, this yields

$$\frac{\mathrm{d}\boldsymbol{g}}{\mathrm{d}t} = B\frac{\mathrm{d}\boldsymbol{f}}{\mathrm{d}t} + \frac{\mathrm{d}\boldsymbol{B}}{\mathrm{d}t}\boldsymbol{f} \tag{19}$$

To proceed further now note that

$$\frac{\mathrm{d}\boldsymbol{f}}{\mathrm{d}t} = \frac{\partial \boldsymbol{f}}{\partial \boldsymbol{x}}\frac{\mathrm{d}\boldsymbol{x}}{\mathrm{d}t} = \boldsymbol{J}\boldsymbol{f} = \boldsymbol{J}\boldsymbol{A}\boldsymbol{g}, \tag{20}$$

with $\boldsymbol{J}$ representing the previously defined Jacobian of the system. Additionally, note that $\boldsymbol{B}\boldsymbol{A} = \boldsymbol{I}$, such that

$$\frac{\mathrm{d}\boldsymbol{B}}{\mathrm{d}t}\boldsymbol{A} + \boldsymbol{B}\frac{\mathrm{d}\boldsymbol{A}}{\mathrm{d}t} = 0. \tag{21}$$

Substituting the results from equations (20), (17), and (21) into (19) yields

$$\dot{\boldsymbol{g}} = \left(\boldsymbol{B}\boldsymbol{J}\boldsymbol{A} - \boldsymbol{B}\frac{\mathrm{d}\boldsymbol{A}}{\mathrm{d}t}\right)\boldsymbol{g} = \boldsymbol{\Lambda}\boldsymbol{g}, \tag{22}$$

providing a system of ODEs for $\boldsymbol{g}$.

If it is assumed that a basis $\boldsymbol{A}$ was selected such that $\boldsymbol{\Lambda}$ is in a block diagonal form such that the ODEs from equation (22) decouple into a system of $\hat{n}$ slow ODEs and $n - \hat{n}$ fast ODEs

$$\begin{pmatrix} \dot{\boldsymbol{g}}_f \\ \dot{\boldsymbol{g}}_s \end{pmatrix} = \begin{pmatrix} \boldsymbol{\Lambda}_f & 0 \\ 0 & \boldsymbol{\Lambda}_s \end{pmatrix} \begin{pmatrix} \boldsymbol{g}_f \\ \boldsymbol{g}_s \end{pmatrix}. \tag{23}$$

Corresponding to the slow/fast groupings, the basis $\boldsymbol{A}$ and its inverse $\boldsymbol{B}$ can also be partitioned into fast and slow parts, such that

$$\boldsymbol{A} = \begin{pmatrix} \boldsymbol{A}_f & \boldsymbol{A}_s \end{pmatrix}, \text{ and } \boldsymbol{B} = \begin{pmatrix} \boldsymbol{B}_f \\ \boldsymbol{B}_s \end{pmatrix}. \tag{24}$$

Hence, under this change of basis the system is now partitioned as

$$\dot{\boldsymbol{x}}(t) = \boldsymbol{A}_f\boldsymbol{g_f} + \boldsymbol{A}_s\boldsymbol{g_s} = \boldsymbol{f}_f + \boldsymbol{f}_s \tag{25}$$

and taking the assumption $\boldsymbol{f}_f \to 0$ on the timescale of interest allows the use of the simplified system of DAEs

$$\dot{\boldsymbol{x}}(t) \approx \boldsymbol{f}_s,$$
$$\boldsymbol{f}_f \approx 0.$$

Whilst the above derivation demonstrates how a model reduction can be applied for a given basis $\boldsymbol{A}(t)$, it does not provide any instruction on how to select an appropriate choice of this basis for the reduction of the system. To obtain an approximation of the ideal such basis, CSP proposes an iterative process. Beginning

with some initial guess basis $\boldsymbol{A}_0$ and its associated inverse $\boldsymbol{B}_0$, each iteration $\boldsymbol{A}_q$ for $q = 1, 2, \ldots$ brings the system described by the nullclines of the fast ODEs closer to the slow manifold of the model. To obtain this approximation, first note that at the qth iterate the matrix $\boldsymbol{\Lambda}_q$ is decomposed as

$$\boldsymbol{\Lambda}_q = \boldsymbol{B}_q \boldsymbol{J} \boldsymbol{A}_q - \boldsymbol{B}_q \frac{\mathrm{d}\boldsymbol{A}_q}{\mathrm{d}t} = \begin{pmatrix} \boldsymbol{\Lambda}_{11q} & \boldsymbol{\Lambda}_{12q} \\ \boldsymbol{\Lambda}_{21q} & \boldsymbol{\Lambda}_{22q} \end{pmatrix}. \tag{26}$$

The following can be used as update criteria for the basis

$$\begin{aligned} \boldsymbol{A}_{q+1} &= \boldsymbol{A}_q \left(\boldsymbol{I} - \boldsymbol{U}_q\right)\left(\boldsymbol{I} + \boldsymbol{L}_q\right) \\ \boldsymbol{B}_{q+1} &= \left(\boldsymbol{I} - \boldsymbol{L}_q\right)\left(\boldsymbol{I} + \boldsymbol{U}_q\right)\boldsymbol{B}_q \end{aligned}$$

where $\boldsymbol{U}_q$ and $\boldsymbol{L}_q$ represent the matrices given by

$$\boldsymbol{U}_q = \begin{pmatrix} 0 & \left(\boldsymbol{\Lambda}_{11q}\right)^{-1} \boldsymbol{\Lambda}_{12q} \\ 0 & 0 \end{pmatrix}, \ \boldsymbol{L}_q = \begin{pmatrix} 0 & 0 \\ \boldsymbol{\Lambda}_{21q} \left(\boldsymbol{\Lambda}_{11q}\right)^{-1} & 0 \end{pmatrix}. \tag{27}$$

In the linear case the ideal basis would be the ordered eigenbasis. In the nonlinear case the ideal basis will be time-varying, however the eigenbasis serves as a common choice for the trial basis $\boldsymbol{A}_0$.

The 'ideal' basis obtained iteratively by CSP can also be used to obtain 'CSP output data' - this is a set of information on the system's species and reactions that is significant for model analysis and can, possibly, be used to guide the more traditional coordinate preserving methods based upon singular perturbation. In particular the method provides three indices - the radical pointer which can be used to identify whether any species are potentially in QSSA, the participation index which provides a means to determine if any reactions are potentially in rapid equilibrium, and the importance index which is strongly related to the sensitivity indices that are discussed in the sensitivity analysis section.

1.5 Local sensitivity analysis

Recall from the main text that the aim of local sensitivity analysis is to construct a sensitivity matrix $R(t)$ such that the entries $r_{ij}(t)$ describe the normalised effect of perturbing the jth rate parameter on the ith state-variable, this can hence be defined as

$$\boldsymbol{R}(t) = \left\{ r_{ij}(t) = \left. \frac{\partial \log\left(x_i(\boldsymbol{p}, t)\right)}{\partial \log\left(p_j\right)} \right|_{\boldsymbol{p}=\boldsymbol{p}^*} \right\}. \tag{28}$$

Note that this can be rewritten as

$$r_{ij}(t) = \left. \frac{\partial x_i(\boldsymbol{p}, t)}{\partial p_j} \frac{p_j}{x_i(\boldsymbol{p}, t)} \right|_{\boldsymbol{p}=\boldsymbol{p}^*} = \left. \frac{\partial x_i(\boldsymbol{p}, t)}{\partial p_j} \right|_{\boldsymbol{p}=\boldsymbol{p}^*} \frac{p_j^*}{x_i(\boldsymbol{p}^*, t)}. \tag{29}$$

There exist a number of numerical approaches for computing numerical values for the partial derivative defined in equation (29) — the most simple approach is through a finite difference approximation of the form

$$\frac{\partial x_i(\boldsymbol{p})}{\partial p_j} \approx \frac{x_i(p_j^* + \Delta p_j) - x_i(p_j^*)}{\Delta p_j}. \tag{30}$$

The accuracy of this approximation will depend upon the size of the perturbation Δp_j employed and the points $\boldsymbol{x}(t, \boldsymbol{p})$ at which this difference is evaluated. It is common to take repeated perturbations at multiple trajectory points and average the estimated sensitivity coefficients so as to obtain more robust approximations.

Alternatively, the sensitivity coefficients can be estimated by solving a system of associated sensitivity ODEs. These ODEs can be obtained by noting that

$$\begin{aligned}\frac{\mathrm{d}}{\mathrm{d}t}\frac{\partial \boldsymbol{x}(t)}{\partial \boldsymbol{p}} &= \frac{\partial}{\partial \boldsymbol{p}}\left[\frac{\mathrm{d}\boldsymbol{x}(t)}{\mathrm{d}t}\right], \\ &= \frac{\mathrm{d}}{\mathrm{d}\boldsymbol{p}}\left[\boldsymbol{S}\boldsymbol{v}(\boldsymbol{x},\boldsymbol{p})\right], \\ &= \boldsymbol{S}\left(\frac{\partial \boldsymbol{v}(\boldsymbol{x},\boldsymbol{p})}{\partial \boldsymbol{x}}\frac{\partial \boldsymbol{x}}{\partial \boldsymbol{p}} + \frac{\partial \boldsymbol{v}(\boldsymbol{x},\boldsymbol{p})}{\partial \boldsymbol{p}}\right).\end{aligned}$$

Hence, defining $\boldsymbol{z}(t) = \frac{\partial \boldsymbol{x}(t)}{\partial \boldsymbol{p}}$ and recalling equations (48) and (49) yields the system of ODEs

$$\frac{\mathrm{d}\boldsymbol{z}(t)}{\mathrm{d}t} = \boldsymbol{J}\boldsymbol{z}(t) + \boldsymbol{S}\frac{\partial \boldsymbol{v}(\boldsymbol{x},\boldsymbol{p})}{\partial \boldsymbol{p}} \tag{31}$$

which is typically numerically simulated under the initial conditions $\boldsymbol{z}(0) = 0$. Unlike the finite difference approach, approximations obtained via the solution of these ODEs are not dependent on the perturbation sizes sampled. They remain dependent, however, on the point $\boldsymbol{x}_c = \boldsymbol{x}(t)$ and $\boldsymbol{p}^* = \boldsymbol{p}$ at which the Jacobian J and the partial derivative $\frac{\partial \boldsymbol{v}(\boldsymbol{x},\boldsymbol{p})}{\partial \boldsymbol{p}}$ are evaluated. Solving this system of ODEs can also be made more computational efficient by employing Green's function matrix [3].

2 A nonlinear example system

Consider the nonlinear example defined by the set of chemical equations

$$\begin{aligned} A + B &\underset{k_2}{\overset{k_1}{\rightleftarrows}} AB \overset{k_3}{\to} C + B, \\ C + D &\underset{k_5}{\overset{k_4}{\rightleftarrows}} CD, \\ C + E &\underset{k_7}{\overset{k_6}{\rightleftarrows}} CE \overset{k_8}{\to} A + E, \\ A + U &\overset{k_9}{\to} U + F, \\ F &\overset{k_{10}}{\to} A, \end{aligned}$$

This system possesses 9 species, 6 reactions, 10 kinetic rate constants, and 1 input U. Whilst this is only a contrived example, it could be considered to represent the enzyme B catalysing the transformation of a substrate A to the form C. Here, C in turn can bind with E to revert to form A or can bind with D to undergo degradation, and U represents an input molecule that catalyzes the transformation of species A to species F — hence sequestering the substrate from performing the autonomous process described above. This system is depicted schematically in Figure 1.

This set of chemical equations can be modelled as dynamical system of ODEs via the Law of Mass Action, such that

$$\dot{\boldsymbol{x}}(t) = S\boldsymbol{v}\left(\boldsymbol{x}(t),\boldsymbol{k}\right), \tag{32a}$$

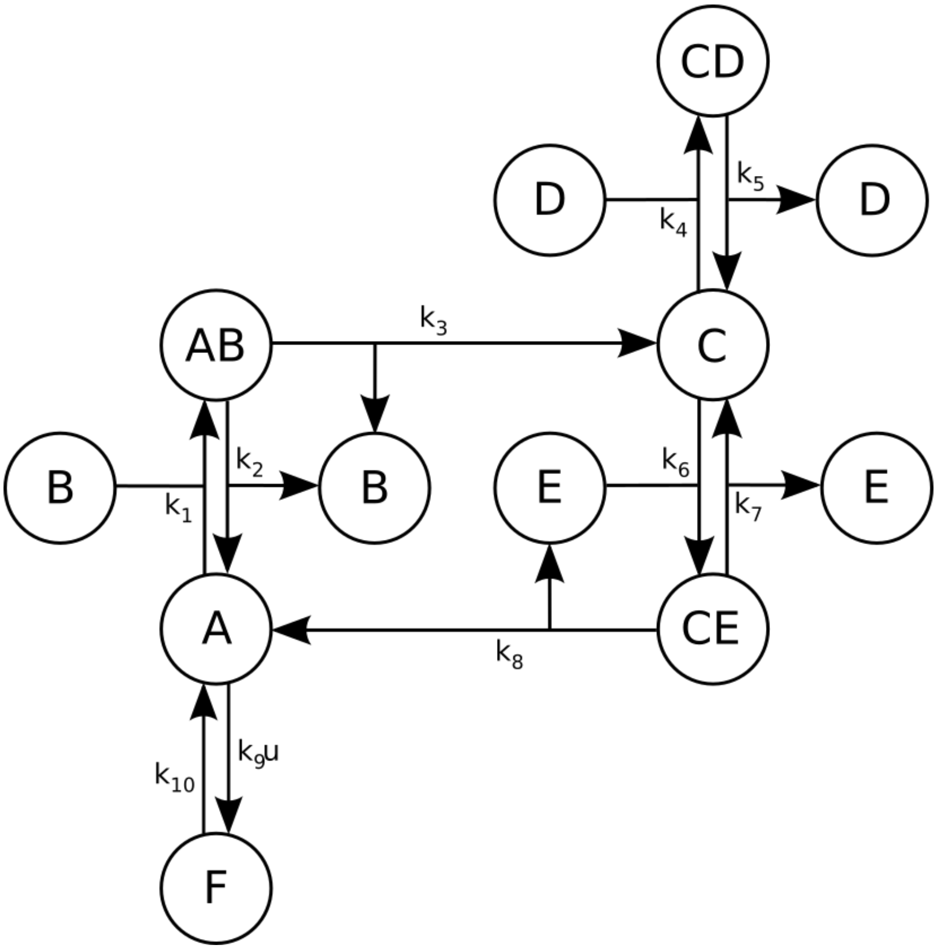

Figure 1. A nonlinear, toy-example system for the demonstration of model reduction methodologies.

with

$$S = \begin{pmatrix} 1 & 0 & 0 & 0 & 1 & -1 & 1 \\ 1 & 1 & 0 & 0 & 0 & 0 & 0 \\ -1 & -1 & 0 & 0 & 0 & 0 & 0 \\ 0 & 1 & 1 & 1 & 0 & 0 & 0 \\ 0 & 0 & -1 & 0 & 0 & 0 & 0 \\ 0 & 0 & 1 & 0 & 0 & 0 & 0 \\ 0 & 0 & 0 & 1 & 1 & 0 & 0 \\ 0 & 0 & 0 & -1 & -1 & 0 & 0 \\ 0 & 0 & 0 & 0 & 0 & 1 & -1 \end{pmatrix}, \quad \boldsymbol{v} = \begin{pmatrix} k1\,x3 - k2\,x1\,x2 \\ k3\,x3 \\ k5\,x5 - k4\,x4\,x6 \\ k7\,x8 - k6\,x4\,x7 \\ k8\,x8 \\ U\,k9\,x1 \\ k10\,x9 \end{pmatrix}. \tag{32b}$$

where the state-variables have been defined as $[A] = x_1(t)$, $[B] = x_2(t)$, $[AB] = x_3(t)$, $[C] = x_4(t)$, $[CD] = x_5(t)$, $[D] = x_6(t)$, $[E] = x_7(t)$, $[CE] = x_8(t)$, and $[F] = x_9(t)$. Additionally, u is defined as the concentration of the input molecule U, such that $[U] = u$ which is assumed here to be time invariant.

For the sake of our examples, let us define a single output $y = x_6(t) = [CD]$. This system can then clearly be expressed in a state-space representation form

$$\begin{aligned} \dot{\boldsymbol{x}}(t) &= \boldsymbol{f}\,(\boldsymbol{x}, t) + \boldsymbol{g}\,(\boldsymbol{x}, t)\,u, \\ y &= x_6(t). \end{aligned}$$

As discussed in the main text, many methods of model reduction require full model parameterisation. For the model here a highly simplified parameterisation is selected and is represented in Table 1. Additionally, the initial conditions are selected such that $x_1(0) = x_4(0) = 1.54$, $x_2(0) = x_7(0) = 0.566$, $x_3(0) = x_8(0) = 0.435$, $x_4(0) = 1.54$, $x_5(0) = 6.06$, $x_6(0) = 3.94$, and $x_9(0) = 0$, which represents the steady-state of the system under the condition $U = 0$.

2.1 Conservation analysis

Now turning to conservation analysis and following the ideas outlined by Reder [4], the existence of conservation relations in a model implies that

$$\Gamma\dot{\boldsymbol{x}}(t) = 0 \tag{33}$$

where Γ is the $h \times n$ conservation matrix – the rows represent the linear combinations of species that are constant in time. By integration,

$$\Gamma\boldsymbol{x}(t) = \boldsymbol{c}, \tag{34}$$

the h individual elements of which are known as conservation relations, with $\boldsymbol{c} \in \mathbb{R}^h$ representing a set of constants known as conserved values.

Solving these conservation relations for specific species and substituting into the original model, yields a system of differential algebraic equations (DAEs). To achieve this partition $\boldsymbol{x}$ into two subsets: $\boldsymbol{x}_d$ an h dimensional subset of the species with each element corresponding to a single species involved in a given conservation relation termed the dependent species. And $\boldsymbol{x}_i$ an $n - h$ dimensional subset accommodating all remaining state-variables, termed the independent species, such that

$$\boldsymbol{x}(t) = \begin{bmatrix} \boldsymbol{x}_d(t) \\ \boldsymbol{x}_i(t) \end{bmatrix}. \tag{35}$$

Then from equation (34)

$$\Gamma \begin{bmatrix} \boldsymbol{x}_d(t) \\ \boldsymbol{x}_i(t) \end{bmatrix} = \boldsymbol{c}. \tag{36}$$

Table 1. The set of parameter values associated with the nonlinear example model as defined by equation (32).

Parameter	Value	Units
k_1	1	$(\mu M \cdot s)^{-1}$
k_2	1	s^{-1}
k_3	1	s^{-1}
k_4	1	$(\mu M \cdot s)^{-1}$
k_5	1	s^{-1}
k_6	1	$(\mu M \cdot s)^{-1}$
k_7	1	s^{-1}
k_8	1	s^{-1}
k_9	100	$(\mu M \cdot s)^{-1}$
k_{10}	1	s^{-1}
B_T	1	μM
D_T	10	μM
E_T	1	μM
S_T	10	μM

This is a system of linear equations and hence if Γ is expressed in reduced row echelon form, such that

$$\Gamma = [I_h \; N_0], \tag{37}$$

with I_h representing the h dimensional identity matrix and N_0 a $h \times (n-h)$ matrix, it becomes apparent that

$$\boldsymbol{x}_d(t) = \boldsymbol{c} - N_0 \boldsymbol{x}_i(t). \tag{38}$$

This implies that the subset of dependent species $\boldsymbol{x}_d$ can be eliminated from the governing system of ODEs by substituting in the appropriate element of equation (38). Hence, given the stoichiometric model form, a system exhibiting conservation relations can be expressed in the form of a semi-explicit system of DAEs, such that

$$\dot{\boldsymbol{x}}_i = S_i \boldsymbol{v}(\boldsymbol{x}_i(t)), \tag{39a}$$

$$\boldsymbol{x}_d(t) = N_0 \boldsymbol{x}_i(t) - \boldsymbol{c}, \tag{39b}$$

where equation (39b) has been exploited in equation (39a) to obtain a system of ODEs such that state-variables $\boldsymbol{x}_d$ are no longer explicitly given. Additionally, S_i here represents the rows of the stoichiometric matrix corresponding to the independent state-variables $\boldsymbol{x}_i$.

Obtaining the conservation matrix Γ, particularly for large systems, is often not feasible from simple inspection. To understand a more algorithmic approach for obtaining this matrix, begin by recalling the stoichiometric form of a model. Decomposing the stoichiometric matrix via the same partition as the set of species leads to the system

$$\begin{pmatrix} \dot{\boldsymbol{x}}_d(t) \\ \dot{\boldsymbol{x}}_i(t) \end{pmatrix} = \begin{pmatrix} S_d \\ S_i \end{pmatrix} \boldsymbol{v}(\boldsymbol{x}_d(t), \boldsymbol{x}_i(t)). \tag{40}$$

However, via differentiation of equation (38)

$$\dot{\boldsymbol{x}}_d(t) = -N_0 \dot{\boldsymbol{x}}_i(t) = -N_0 S_i \boldsymbol{v}(\boldsymbol{x}_d(t), \boldsymbol{x}_i(t)). \tag{41}$$

Hence, $S_d = -N_0 S_i$ and therefore each conservation relation can be seen as corresponding to a linear dependency in the stoichiometry matrix. As such, conservation relations can be found by seeking the left null space Z_n of S (i.e. via finding the null space of S^T) such that

$$Z_n = \{ \boldsymbol{z} \in \mathbb{R}^n \,|\, S^{\intercal} \boldsymbol{z} = \boldsymbol{0} \}, \tag{42}$$

and hence $Z_n^{\intercal} S = \boldsymbol{0}$. This implies that

$$Z_n^{\intercal} S \boldsymbol{v}\left(\dot{\boldsymbol{x}}(t)\right) = \boldsymbol{0} = Z_n^{\intercal} \dot{\boldsymbol{x}}(t) \tag{43}$$

and therefore via comparison to equation (33) it is clear that

$$Z_n^{\intercal} = \Gamma, \tag{44}$$

such that the conservation matrix is equal to the transpose of the left null space of the stoichiometry matrix.

In the case of our example system represented by equation (32), calculation of the left null-space yields

$$Z_n = \begin{pmatrix} 0 & 0 & 0 & 1 \\ 1 & 0 & 0 & 0 \\ 1 & 0 & 0 & 1 \\ 0 & 0 & 0 & 1 \\ 0 & 1 & 0 & 1 \\ 0 & 1 & 0 & 0 \\ 0 & 0 & 1 & 0 \\ 0 & 0 & 1 & 1 \\ 0 & 0 & 0 & 1 \end{pmatrix}, \tag{45}$$

implying that we have the conservation relations

$$B_T = x_2(t) + x_3(t), \tag{46a}$$
$$D_T = x_5(t) + x_6(t), \tag{46b}$$
$$E_T = x_7(t) + x_8(t), \tag{46c}$$
$$S_T = x_1(t) + x_3(t) + x_4(t) + x_5(t) + x_8(t) + x_9(t) \tag{46d}$$

Finally, substituting these conservation relations into the system yields the simplified realisation,

$$\frac{\mathrm{d}x_1(t)}{\mathrm{d}t} = k_1 x_3(t) - k_{10}\left(x_1(t) - S_T - C_T + x_3(t) + x_4(t) + x_5(t) + x_8(t)\right) + k_8 x_8(t) - U\, k_9 x_1(t) - k_2 x_1(t)\, \left(B_T - x_3(t)\right), \tag{47a}$$
$$\frac{\mathrm{d}x_3(t)}{\mathrm{d}t} = k_2 x_1(t)\, \left(B_T - x_3(t)\right) - k_3 x_3(t) - k_1 x_3(t), \tag{47b}$$
$$\frac{\mathrm{d}x_4(t)}{\mathrm{d}t} = k_3 x_3(t) + k_5 x_5(t) + k_7 x_8(t) - k_4 x_4(t)\, \left(C_T - x_5(t)\right) - k_6 x_4(t)\, \left(E_T - x_8(t)\right), \tag{47c}$$
$$\frac{\mathrm{d}x_5(t)}{\mathrm{d}t} = k_4 x_4(t)\, \left(C_T - x_5(t)\right) - k_5 x_5(t), \tag{47d}$$
$$\frac{\mathrm{d}x_8(t)}{\mathrm{d}t} = k_6 x_4(t)\, \left(E_T - x_8(t)\right) - k_8 x_8(t) - k_7 x_8(t). \tag{47e}$$

2.2 Model Linearisation

Certain methods also require that the system they are applied to is linear. For nonlinear systems we therefore require the system to first be linearised for the application of such methods. As discussed in the main text, systems of ODEs can be linearised around a given state $\boldsymbol{x}_c$ by calculating the Jacobian matrix

$$\boldsymbol{J}_{\boldsymbol{x}_c} = \left.\boldsymbol{S}\boldsymbol{E}\right|_{\boldsymbol{x}(t)=\boldsymbol{x}_c}. \tag{48}$$

Here, the matrix $\boldsymbol{E}$ is referred to as the elasticity matrix, with entries

$$\boldsymbol{E} = \left\{ e_{ij} = \frac{\partial v_i\left(\boldsymbol{x}, \boldsymbol{p}\right)}{\partial x_j} \right\}. \tag{49}$$

Then, via a first order Taylor expansion, the system can be approximated in the neighbourhood of $\boldsymbol{x}_c$ by

$$\dot{\boldsymbol{x}}(t) \approx \boldsymbol{S}\boldsymbol{v}\left(\boldsymbol{x}_c, \boldsymbol{p}\right) + \boldsymbol{J}_{\boldsymbol{x}_c}\left(\boldsymbol{x}(t) - \boldsymbol{x}_c\right). \tag{50}$$

Evaluated at the system's previously defined initial condition, calculating the Jacobian for this system yields

$$J_{\boldsymbol{x}_0} = \begin{pmatrix} -1.57 - 100U & 1.54 & -1 & -1 & 0 \\ 0.566 & -3.54 & 0 & 0 & 0 \\ 0 & 1 & -4.51 & 2.54 & 2.54 \\ 0 & 0 & 3.94 & -2.54 & 0 \\ 0 & 0 & 0.566 & 0 & -3.54 \end{pmatrix}. \tag{51}$$

2.3 Coordinate Preserving Timescale Exploitation

Here we seek to apply the QSSA for the reduction of the nonlinear example model previously introduced. Whilst in the main paper we review a number of published methods for ascertaining the species that can be eliminated through application of the QSSA, in this instance it is a reasonable that the complex species AB and CE are likely to evolve on a fast timescale for a large range of biologically reasonable parameterisations. Often, in realistic systems, these complexes will be transitional steps in reactions where enzymes B and E are catalyzing some substrate protein.

Mathematically, the QSSA is then equivalent to assuming that Equations (47b) and (47e) are approximately at zero after a very short initial transient period, such that

$$\begin{aligned} k_2x_1(t)\,\left(B_T - x_3(t)\right) - k_3x_3(t) - k_1x_3(t) &\approx 0, \\ k_6x_4(t)\,\left(E_T - x_8(t)\right) - k_8x_8(t) - k_7x_8(t) &\approx 0, \end{aligned}$$

which implies we can take the approximations

$$\begin{aligned} x_3(t) &\approx \frac{k_2x_1(t)B_T}{k_1 + k_2x_1(t) + k_3}, \\ x_8(t) &\approx \frac{k_6x_4(t)E_T}{k_6x_4 + k_7 + k_8}. \end{aligned}$$

Finally, substituting these approximations into system represented by Equation (47) yields the reduced, 3 dimensional system

$$\begin{aligned} \frac{\mathrm{d}x_1(t)}{\mathrm{d}t} &= \frac{k_1k_2x_1(t)B_T}{k_1 + k_2x_1(t) + k_3} \\ &\quad - k_{10}\left(x_1(t) - S_T - C_T + \frac{k_2x_1(t)B_T}{k_1 + k_2x_1(t) + k_3} + x_4(t) + x_5(t) + \frac{k_6x_4(t)E_T}{k_6x_4 + k_7 + k_8}\right) \\ &\quad + \frac{k_8k_6x_4(t)E_T}{k_6x_4 + k_7 + k_8} - U\,k_9x_1(t) - k_2x_1(t)\left(B_T - \frac{k_2x_1(t)B_T}{k_1 + k_2x_1(t) + k_3}\right), && (52a) \\ \frac{\mathrm{d}x_4(t)}{\mathrm{d}t} &= \frac{k_3k_2x_1(t)B_T}{k_1 + k_2x_1(t) + k_3} + k_5x_5(t) + \frac{k_7k_6x_4(t)E_T}{k_6x_4 + k_7 + k_8} \\ &\quad - k_4x_4(t)\,\left(C_T - x_5(t)\right) - k_6x_4(t)\left(E_T - \frac{k_6x_4(t)E_T}{k_6x_4 + k_7 + k_8}\right), && (52b) \\ \frac{\mathrm{d}x_5(t)}{\mathrm{d}t} &= k_4x_4(t)\,\left(C_T - x_5(t)\right) - k_5x_5(t). && (52c) \end{aligned}$$

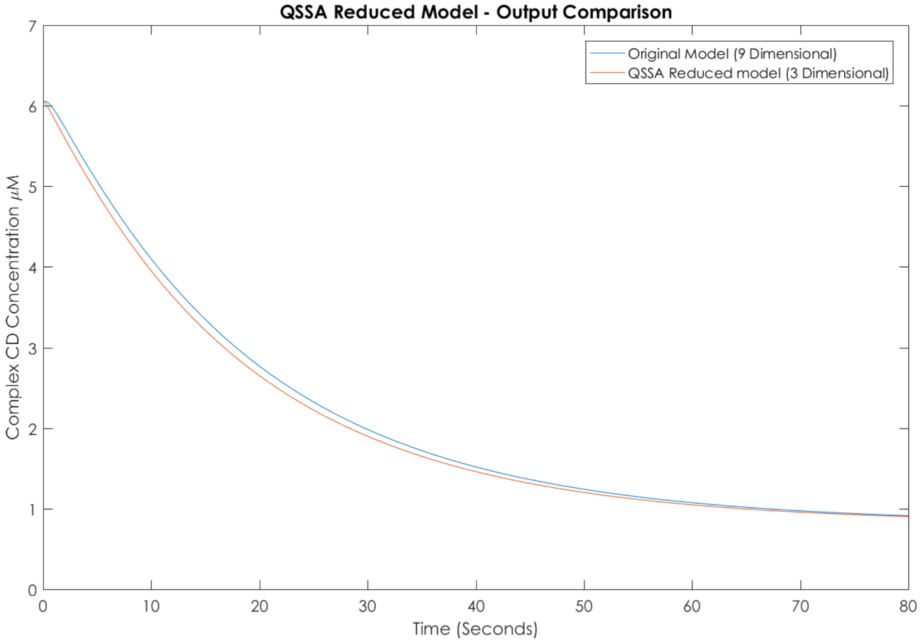

Figure 2. Model reduction via quasi steady-state analysis. Comparison of result for the output variable $[CD]$ for the reduced 3 dimensional model, vs the original 9 dimensional system.

Using the previously defined model parameterisation and initial condition, and setting $U = 1$, it is possible to simulate this reduced model. Figure 2 shows how the reduced model compares to the original system for the simulation of our assigned output – the concentration of the complex CD.

2.4 Coordinate Transforming Timescale Exploitation

In this section we seek to apply the intrinsic low dimensional manifold (ILDM) method introduced in the main text for the reduction of the nonlinear example model outlined above. Here we begin with the Jacobian $J_{\boldsymbol{x}_0}$ defined by equation (51) and set the input $U = 1$. We then seek to apply a Schur decomposition to yield

$$\boldsymbol{V} = \boldsymbol{Q}^{-1} J_{\boldsymbol{x}_0} \boldsymbol{Q} = \begin{bmatrix} \boldsymbol{V}_s & \boldsymbol{V}_c \\ 0 & \boldsymbol{V}_f \end{bmatrix}. \tag{53}$$

In our case, this gives

$$\boldsymbol{V} = \begin{pmatrix} -102 & -0.0633 & -1.40 & 0.0815 & -0.875 \\ 0 & -11.2 & -7.96 & -0.257 & 0.667 \\ 0 & 0 & -0.05037 & -0.787 & 0.607 \\ 0 & 0 & 0 & -2.04 & -0.0465 \\ 0 & 0 & 0 & 0 & -2.09 \end{pmatrix}, \tag{54}$$

$$\boldsymbol{Q} = \begin{pmatrix} 0.00959 & -0.0000757 & 0.00636 & 0.103 & -0.995 \\ -1 & 0.0000829 & 0.000124 & 0.000980 & -0.00954 \\ -0.000104 & -0.739 & -0.671 & 0.0513 & 0.00107 \\ 0.00000941 & 0.669 & -0.741 & -0.0586 & -0.0108 \\ 0.000000996 & 0.0778 & -0.00971 & 0.992 & 0.102 \end{pmatrix} \tag{55}$$

We note that there is an eigengap between the 2nd and 3rd ordered eigenvalues of this system. As a result we choose to construct a 3 dimensional reduced system.

These matrices can be reordered in terms of their eigenvalues to yield a system of the form of represented by equation (53) via the use of Givens rotations. If we let the block upper triangular form $\boldsymbol{U}_f$ corresponds to the fast eigenvalues, $\boldsymbol{U}_s$ to the slow eigenvalues, and $\boldsymbol{U}_c$ refers to the block coupling the fast and slow dynamics of the system under the Schur basis, we have

$$\boldsymbol{V}_f = \begin{pmatrix} -102 & -0.694 \\ 0 & -11.2 \end{pmatrix} \tag{56}$$

$$\boldsymbol{V}_s = \begin{pmatrix} -0.0503 & -1.07 & 0.876 \\ 0 & -2.04 & -0.0468 \\ 0 & 0 & -2.09 \end{pmatrix} \tag{57}$$

$$\boldsymbol{V}_c = \begin{pmatrix} -1.11 & -7.86 \\ 0.134 & -0.674 \\ -0.945 & 0.883 \end{pmatrix}. \tag{58}$$

If we now seek to decouple the slow and fast terms of the Jacobian (i.e. eliminate the $\boldsymbol{U}_c$ terms), then we seek to compute the solution $\boldsymbol{Y}$ of the Sylvester equation

$$\boldsymbol{U}_{\text{slow}}\boldsymbol{Y} - \boldsymbol{Y}\boldsymbol{U}_{\text{fast}} = \boldsymbol{U}_{\text{coup}}. \tag{59}$$

In this case, this gives

$$\boldsymbol{Y} = \begin{pmatrix} 0.0108 & 0.722 \\ -0.00133 & 0.0736 \\ 0.00946 & -0.0982 \end{pmatrix}. \tag{60}$$

It is then possible to construct the transformation T of the state variables which decouples the fast and slow components of the system via

$$\boldsymbol{T} = \boldsymbol{Q}\left(\boldsymbol{I} + \begin{bmatrix} 0 & \boldsymbol{Y} \\ 0 & 0 \end{bmatrix}\right) \quad \text{and} \quad \boldsymbol{T}^{-1} = \left(I - \begin{bmatrix} 0 & \boldsymbol{Y} \\ 0 & 0 \end{bmatrix}\right)\boldsymbol{Q}^{-1}, \tag{61}$$

and hence obtain

$$\boldsymbol{T}^{-1}\boldsymbol{J}_{\boldsymbol{x}_c}\boldsymbol{T} = \begin{bmatrix} \boldsymbol{U}_{\text{slow}} & 0 \\ 0 & \boldsymbol{U}_{\text{fast}} \end{bmatrix}. \tag{62}$$

For our nonlinear example model this gives

$$T = \begin{pmatrix} 0.00511 & 0.102 & -0.989 & -0.00959 & 0.0000289 \\ 0.0109 & -0.000364 & -0.0000125 & 1 & 0.00668 \\ -0.115 & 0.124 & -0.0952 & 0.000104 & 0.917 \\ -0.992 & -0.0666 & -0.000238 & -0.00000941 & -0.829 \\ -0.0532 & 0.985 & 0.115 & -0.000000996 & -0.0965 \end{pmatrix}, \tag{63}$$

$$T^{-1} = \begin{pmatrix} 0.0833 & 0.000876 & -0.823 & -0.914 & 0.0329 \\ 0.110 & 0.00105 & 0.0515 & -0.0587 & 0.994 \\ -1 & -0.00959 & 0.00107 & -0.0109 & 0.103 \\ -0.000154 & 1 & 0.00241 & 0.0106 & 0.000802 \\ -0.108 & -0.00114 & 0.981 & -0.107 & -0.119 \end{pmatrix}. \tag{64}$$

Via the Petrov-Galerkin projection we can then construct the reduced system

$$\dot{\bar{\boldsymbol{x}}}(t) = \boldsymbol{T}^{-1}\boldsymbol{S}\boldsymbol{v}\left(\boldsymbol{T}\bar{\boldsymbol{x}}(t), \boldsymbol{k}\right). \tag{65}$$

Hence, on the slow timescale we have the reduced 3 dimensional system

$$\begin{aligned} \frac{\mathrm{d}\bar{x}_1}{\mathrm{d}t} &= 0.051\bar{x}_1(t)^2 - 0.014U - 1.050\bar{x}_2(t)^2 + 0.093\bar{x}_3(t)^2 - 0.001U\bar{x}_1(t) - 0.072\bar{x}_1(t) \\ &\quad - 1.591\bar{x}_2(t) + 0.945\bar{x}_3(t) + 0.917\bar{x}_1(t)\bar{x}_2(t) + 0.164\bar{x}_1(t)\bar{x}_3(t) + 0.687\bar{x}_2(t)\bar{x}_3(t) + 0.013, \end{aligned} \tag{66}$$

$$\begin{aligned} \frac{\mathrm{d}\bar{x}_2}{\mathrm{d}t} &= 0.068\bar{x}_1(t)^2 - 0.017U - 1.16\bar{x}_2(t)^2 + 0.103\bar{x}_3(t)^2 - 0.001U\bar{x}_1(t) - 0.028\bar{x}_1(t) \\ &\quad - 2.617\bar{x}_2(t) + 0.059\bar{x}_3(t) + 0.997\bar{x}_1(t)\bar{x}_2(t) + 0.192\bar{x}_1(t)\bar{x}_3(t) + 0.757\bar{x}_2(t)\bar{x}_3(t) + 0.015, \end{aligned} \tag{67}$$

$$\begin{aligned} \frac{\mathrm{d}\bar{x}_3}{\mathrm{d}t} &= 0.154U + 0.008\bar{x}_1(t)^2 - 0.125\bar{x}_2(t)^2 + 0.011\bar{x}_3(t)^2 + 0.010U\bar{x}_1(t) - 0.006\bar{x}_1(t) \\ &\quad + 0.094\bar{x}_2(t) - 3.58\bar{x}_3(t) + 0.119\bar{x}_1(t)\bar{x}_2(t) - 0.087\bar{x}_1(t)\bar{x}_3(t) + 0.085\bar{x}_2(t)\bar{x}_3(t) - 0.173. \end{aligned} \tag{68}$$

Using the previously defined model parameterisation and initial condition, and setting $U = 1$, it is possible to simulate this reduced model. Figure 3 shows how the reduced model compares to the original system for the simulation of our assigned output – the time-varying concentration of the complex CD.

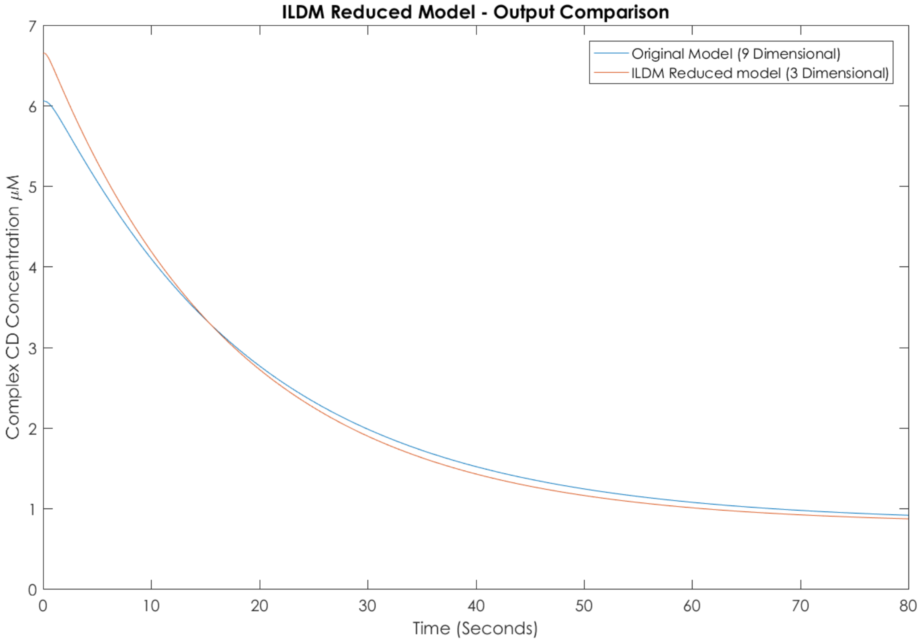

Figure 3. Model reduction via the intrinsic low dimensional manifold method. Comparison of result for the output variable $[CD]$ for the reduced 3 dimensional model, vs the original 9 dimensional system.

2.5 Sensitivity Analysis

In this section we demonstrate the application of sensitivity analysis for the study and reduction of the nonlinear example defined by equation (32). Following the approach outlined in Section 1.5, we begin by computing the normalised sensitivity matrix $\boldsymbol{R}$ for the system, such that

$$\boldsymbol{R}(t) = \left\{ r_{ij}(t) = \frac{\partial \log\left(x_i(\boldsymbol{p}, t)\right)}{\partial \log\left(p_j\right)}\bigg|_{\boldsymbol{p}=\boldsymbol{p}^*} \right\}. \tag{69}$$

Which can be rewritten as

$$r_{ij}(t) = \frac{\partial x_i(\boldsymbol{p}, t)}{\partial p_j}\bigg|_{\boldsymbol{p}=\boldsymbol{p}^*} \frac{p_j^*}{x_i(\boldsymbol{p}^*, t)} \tag{70}$$

describing the effect of perturbing the jth rate parameter on the ith state-variable.

The most common computational approach to calculating the remaining partial derivative is via a finite difference approximation yielding

$$\frac{\partial x_i(\boldsymbol{p}, t)}{\partial p_j} \approx \frac{x_i(p_j^* + \Delta p_j, t) - x_i(p_j^*)}{\Delta p_j}. \tag{71}$$

The accuracy of this approximation, however, will depend upon the size of the perturbation Δp_j employed and the time-points at which the differences are evaluated. It is common to take repeated perturbations at multiple trajectory points and average the estimated sensitivity coefficients so as to obtain more robust approximations.

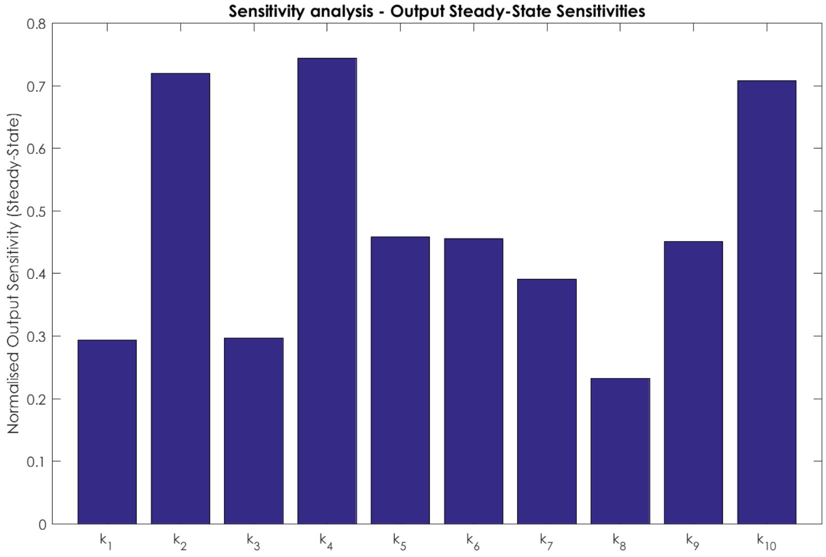

Figure 4. Normalised sensitivity values for the output species $[CD]$.

Focussing only on the steady-state sensitivity of the nonlinear example defined by equation (32), we obtain the normalised sensitivity matrix

$$\boldsymbol{R}_{ss} = \begin{pmatrix} 0.033 & -0.081 & -0.030 & 0.041 & -0.066 & -0.040 & 0.046 & 0.026 & -0.472 & 0.823 \\ 0.014 & -0.035 & 0.015 & -0.002 & 0.003 & 0.002 & -0.002 & -0.001 & 0.021 & -0.034 \\ -0.302 & 0.774 & -0.343 & 0.039 & -0.064 & -0.038 & 0.044 & 0.025 & -0.462 & 0.761 \\ -0.312 & 0.838 & 0.332 & 0.041 & -0.066 & 0.441 & -0.477 & -0.248 & -0.472 & 0.823 \\ -0.294 & 0.720 & 0.296 & -0.458 & 0.744 & 0.391 & -0.456 & -0.232 & -0.451 & 0.708 \\ 0.026 & -0.064 & -0.026 & 0.0409 & -0.066 & -0.035 & 0.041 & 0.021 & 0.040 & -0.063 \\ 0.014 & -0.035 & -0.014 & -0.002 & 0.003 & 0.002 & -0.002 & 0.022 & 0.021 & -0.034 \\ -0.302 & 0.774 & 0.313 & 0.039 & -0.064 & -0.038 & 0.044 & -0.488 & -0.462 & 0.761 \\ 0.033 & -0.081 & -0.030 & 0.041 & -0.066 & -0.040 & 0.046 & 0.026 & 0.055 & -0.089 \end{pmatrix}, \tag{72}$$

where $R(t) \to R_{ss}$ as $t \to \infty$. In the case of the output species $[CD]$, this implies we have normalised steady-state sensitivities depicted in Figure 4.

As described in the main text, once a sensitivity matrix has been obtained, principle component analysis provides a straightforward means to assess the relative importance of each species in determining the overall dynamical behaviour of the system. This can be used to guide the application of model reduction methods and eliminate parameters from parameter fitting processes. In the case of the nonlinear system, a singular value decomposition reveals that the system is dominated by 5 principle components. Analysis of each parameter's relative contribution to these components shows that 2 of the parameters - k_5 and k_8 have a limited influence on the overall steady-state behaviour of the system in comparison to the remainder of the

network. This knowledge could be used to therefore guide the reduction of the network through application of the methods such as the rapid equilibrium approximation or to reduce the number of parameters that are required for data fitting..

2.6 Lumping

In this section we demonstrate the application of linear proper lumping for the reduction of the nonlinear example defined by equation (32). As described in the main text, this implies the reduction of the state-variables $\boldsymbol{x}(t) \in \mathbb{R}^n$ to a reduced set $\tilde{\boldsymbol{x}}(t) \in \mathbb{R}^{\hat{n}}$ via some linear projection $\boldsymbol{L} \in \{0,1\}^{\hat{n}\times n}$, where each row of $\boldsymbol{L}$ is pairwise orthogonal. Hence, we are seeking to create new 'lumped' state-variables as proper sums of the originals, with each of the original variables $\boldsymbol{x}(t)$ corresponding to, at most, one of the reduced variables $\tilde{\boldsymbol{x}}(t)$. Once such a lumping matrix $\boldsymbol{L}$ is found a reduced system can be computed via the Petrov-Galerkin projection (as described in the main text).

In the case of the nonlinear example system, we applied the forward selection strategy described by Dokoumetzidis and Aarons [5] and outlined in the main text. Here the algorithm seeks iteratively to lump two state-variables at a time whilst optimising against simulated output error. Beginning with the system already simplified via the application of conservation analysis (represented by equation (47)) this lumping approach led to the results given in Table 2 and depicted in Figure 5. At the three dimensional level, this leads to the reduced system

$$\frac{\mathrm{d}\tilde{x}_1}{\mathrm{d}t} = 0.056k_8\tilde{x}_3 - 0.339k_3\tilde{x}_1 - 1.0k_{10}\left(\tilde{x}_1 - S_T - C_T + \tilde{x}_2 + \tilde{x}_3\right) - 0.661Uk_9\tilde{x}_1, \tag{73}$$

$$\frac{\mathrm{d}\tilde{x}_2}{\mathrm{d}t} = 0.339k_3\tilde{x}_1 + 0.944k_5\tilde{x}_3 + 0.056k_7\tilde{x}_3 - k_6\left(E_T - 0.056\tilde{x}_3\right)\tilde{x}_2 - k_4\left(C_T - 0.944\tilde{x}_3\right)\tilde{x}_2, \tag{74}$$

$$\frac{\mathrm{d}\tilde{x}_3}{\mathrm{d}t} = k_6\left(E_T - 0.056\tilde{x}_3\right)\tilde{x}_2 - 0.056k_7\tilde{x}_3 - 0.056k_8\tilde{x}_3 - 0.944k_5\tilde{x}_3 + k_4\left(C_T - 0.944\tilde{x}_3\right)\tilde{x}_2, \tag{75}$$

with the initial conditions $\tilde{x}_1(0) = 0.1971$, $\tilde{x}_2(0) = 0.1537$, and $\tilde{x}_3(0) = 0.6492$. This reduced system can then be used to approximate the original state-variables by $\boldsymbol{x} \approx \bar{\boldsymbol{L}}\tilde{\boldsymbol{x}}$ with

$$\bar{L} = \begin{pmatrix} 0.339 & 0 & 0 \\ 0.661 & 0 & 0 \\ 0 & 1 & 0 \\ 0 & 0 & 0.944 \\ 0 & 0 & 0.0556 \end{pmatrix}. \tag{76}$$

2.7 Singular value decomposition based methods

In this section we demonstrate the application of balanced truncation for the reduction of the nonlinear example defined by equation (32). As is described in the main text balanced truncation is designed for the reduction of controlled, linear systems in a state-space representation form, typically such systems can be expressed as

$$\begin{aligned} \dot{\boldsymbol{x}} &= \boldsymbol{A}\boldsymbol{x} + \boldsymbol{B}\boldsymbol{u}, \\ \boldsymbol{y} &= \boldsymbol{C}\boldsymbol{x}. \end{aligned}$$

Once such a linear system has been constructed, balanced truncation can be applied by following the detailed steps given in the main text of the article. This process involves first constructing the system

Table 2. The results for lumping the nonlinear example system under the forward selection strategy.

Dimensions	Output Error	Lumped Variables
4	1.67%	$[A]+[AB],\ [C],\ [CD],\ [CE]$
3	2.29%	$[A]+[AB],\ [C],\ [CD]+[CE]$
2	9.53%	$[A]+[AB],\ [C]+[CD]+[CE]$
1	61.89%	$[A]+[AB]+[C]+[CD]+[CE]$

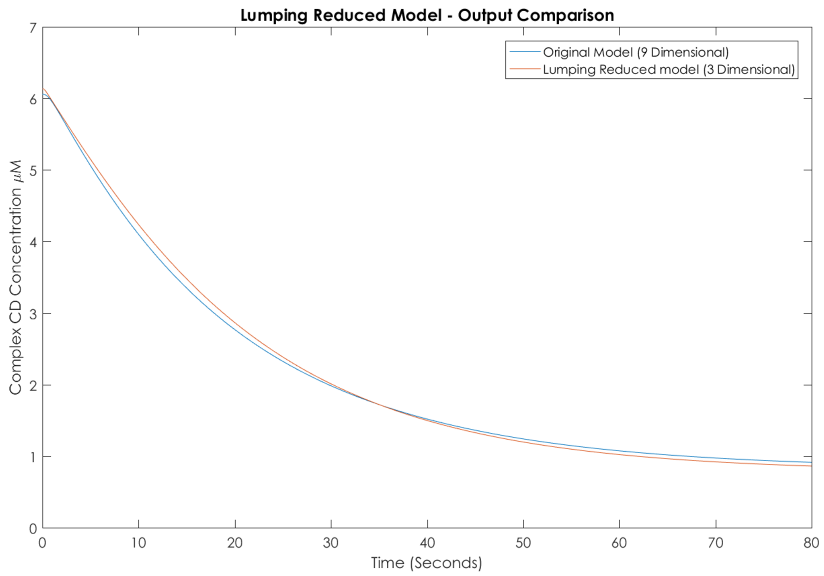

Figure 5. Model reduction via linear proper lumping. Comparison of results for the output variable $[CD]$ for the reduced 3 dimensional model versus the original 9 dimensional system.

controllability and observability Gramians through solving two Lyapunov equations and then seeking a balancing transformation which equalises and diagonalises these Gramians. Using this it is possible to transform the dynamical system over to the new basis governed by the balancing transformation and finally yield a reduced system by truncating those states that are found to be least responsible for driving the system's overall input-output behaviour - corresponding to the smallest singular values of the balanced Gramian.

In the case of our nonlinear example, the system can be expressed in such a form via application of linearisation as in the form of equation (50). Application of the described yields the transformation matrix for the 3 dimensional reduced case of

$$T = \begin{pmatrix} -5.208 & -0.029 & -18.679 & -19.741 & -13.180 \\ -5.557 & -0.032 & -9.123 & 8.636 & -14.053 \\ 1.938 & 0.012 & -20.444 & 12.543 & 4.852 \end{pmatrix} \tag{77}$$

and its generalised inverse

$$\bar{T} = \begin{pmatrix} -0.006 & -0.139 & 0.012 \\ -0.037 & -1.532 & 11.168 \\ -0.019 & 0.006 & -0.033 \\ -0.029 & 0.032 & 0.012 \\ -0.003 & 0.003 & -0.0006 \end{pmatrix}. \tag{78}$$

Hence we can work with the reduced state-variables $\tilde{\boldsymbol{x}}(t) = T\boldsymbol{x}(t)$. Using the Petrov-Galerkin projection we can then use this transformation to yield the reduced 3 dimensional dynamical system

$$\begin{aligned} \frac{\mathrm{d}\tilde{x}_1}{\mathrm{d}t} &= 1.06\,(0.290\tilde{x}_1 - 0.315\tilde{x}_2 - 0.124\tilde{x}_3 + 1)\,(0.0191\tilde{x}_1 - 0.006\tilde{x}_2 + 0.033\tilde{x}_3 + 0.003) \\ &\quad - 5.498\,(0.030\tilde{x}_1 - 0.0291\tilde{x}_2 + 0.006\tilde{x}_3 + 1.093)\,(0.019\tilde{x}_1 - 0.006\tilde{x}_2 + 0.033\tilde{x}_3 + 0.003) \\ &\quad + 5.179\,(0.037\tilde{x}_1 + 1.532\tilde{x}_2 - 11.168\tilde{x}_3 + 48.953)\,(0.059\tilde{x}_1 + 1.393\tilde{x}_2 - 0.125\tilde{x}_3 - 2.057) \\ &\quad - 0.007\tilde{x}_1 + 1.163\tilde{x}_2 + 0.229\tilde{x}_3 - 2.898U\,(0.037\tilde{x}_1 + 1.532\tilde{x}_2 - 11.168\tilde{x}_3 + 48.953) - 4.045, \end{aligned} \tag{79}$$

$$\begin{aligned} \frac{\mathrm{d}\tilde{x}_2}{\mathrm{d}t} &= 4.929\,(0.030\tilde{x}_1 - 0.029\tilde{x}_2 + 0.006\tilde{x}_3 + 1.093)\,(0.019\tilde{x}_1 - 0.006\tilde{x}_2 + 0.033\tilde{x}_3 + 0.003) \\ &\quad - 17.760\,(0.290\tilde{x}_1 - 0.315\tilde{x}_2 - 0.124\tilde{x}_3 + 1)\,(0.019\tilde{x}_1 - 0.006\tilde{x}_2 + 0.033\tilde{x}_3 + 0.003) \\ &\quad + 5.526\,(0.037\tilde{x}_1 + 1.532\tilde{x}_2 - 11.168\tilde{x}_3 + 48.953)\,(0.059\tilde{x}_1 + 1.393\tilde{x}_2 - 0.125\tilde{x}_3 - 2.057) \\ &\quad + 0.443\tilde{x}_1 - 0.829\tilde{x}_2 + 0.144\tilde{x}_3 - 3.150U\,(0.037\tilde{x}_1 + 1.532\tilde{x}_2 - 11.168\tilde{x}_3 + 48.953) - 1.142, \end{aligned} \tag{80}$$

$$\begin{aligned} \frac{\mathrm{d}\tilde{x}_2}{\mathrm{d}t} &= 1.192\tilde{x}_1 - 25.297\,(0.030\tilde{x}_1 - 0.029\tilde{x}_2 + 0.006\tilde{x}_3 + 1.093)\,(0.019\tilde{x}_1 - 0.006\tilde{x}_2 + 0.033\tilde{x}_3 + 0.003) \\ &\quad - 1.926\,(0.037\tilde{x}_1 + 1.532\tilde{x}_2 - 11.168\tilde{x}_3 + 48.953)\,(0.059\tilde{x}_1 + 1.393\tilde{x}_2 - 0.125\tilde{x}_3 - 2.057) \\ &\quad - 32.987\,(0.290\tilde{x}_1 - 0.315\tilde{x}_2 - 0.124\tilde{x}_3 + 1)\,(0.019\tilde{x}_1 - 0.006\tilde{x}_2 + 0.033\tilde{x}_3 + 0.003) \\ &\quad + 2.280\tilde{x}_2 - 0.833\tilde{x}_3 + 1.240U\,(0.037\tilde{x}_1 + 1.532\tilde{x}_2 - 11.168\tilde{x}_3 + 48.953) - 6.540, \end{aligned} \tag{81}$$

with the associated initial conditions $\tilde{x}_1(0) = -15.6324$, $\tilde{x}_2(0) = 2.9729$, and $\tilde{x}_3(0) = 4.7534$. Results for this reduced system are given in Figure 6 which demonstrates the reduced dynamics of the output in the 4, 3, and 2 dimensional reduced cases, respectively. As can be seen from this figure, all 3 reduced models have very similar dynamics and exhibit similar error to the original model — this is due to the fact that the majority of the error was incurred in the initial linearisation step and not through the application of balanced truncation for reduction.

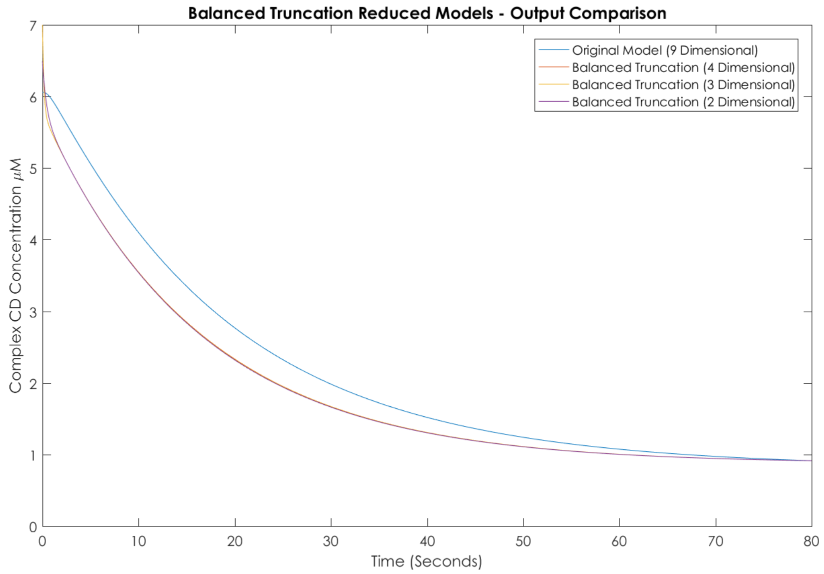

Figure 6. Model reduction via linearisation and balanced truncation. Comparison of results for the output variable $[CD]$ for the reduced 4, 3, and 2 dimensional models vs the original 9 dimensional system. All 3 of the reduced systems are nearly identical in their dynamics — this is due to the fact that the primary cause of error in this reduction is not from the application of Balanced Truncation, but is due to the initial linearisation step.

References

[1] Antoulas, A.C.: Approximation of Large-scale Dynamical Systems. Advances in Design and Control. Society for Industrial and Applied Mathematics, Philadelphia (2005)

[2] Golub, G.H., Van Loan, C.F.: Matrix Computations vol. 3. JHU Press, Maryland, USA (2012)

[3] Rabitz, H., Kramer, M., Dacol, D.: Sensitivity analysis in chemical kinetics. Annual review of physical chemistry **34**(1), 419–461 (1983)

[4] Reder, C.: Metabolic control theory: a structural approach. Journal of Theoretical Biology **135**(2), 175–201 (1988)

[5] Dokoumetzidis, A., Aarons, L.: Proper lumping in systems biology models. IET Systems Biology **3**(1), 40–51 (2009)
